# Projected budget impact of pharmacotherapy scenarios for obesity and related conditions in Peru: A national survey analysis of 76,819 participants

**DOI:** 10.1016/j.obpill.2026.100308

**Published:** 2026-07-25

**Authors:** Victor J. Vera Ponce

**Affiliations:** Facultad de Medicina (FAMED), Universidad Nacional Toribio Rodríguez de Mendoza de Amazonas (UNTRM), Amazonas, Peru

**Keywords:** Budget impact analysis, Liraglutide, National health surveys, Naltrexone/bupropion, Obesity, Obesity medication, Peru, Pharmacotherapy, Semaglutide

## Abstract

**Background:**

The increasing prevalence of obesity is generating demand for chronic pharmacologic treatment, but Latin American evidence on population-level budget implications remains limited.

**Methods:**

This was an observational, cross-sectional descriptive study using ENDES (Encuesta Demográfica y de Salud Familiar or Demographic and Family Health Survey) 2022–2024 data, with a deterministic scenario-based budget impact analysis component. Survey-observable candidate populations and gross medication-acquisition requirements were estimated under the complex survey design. Liraglutide, extended-release naltrexone/bupropion, and extended-release phentermine/topiramate were modeled, while semaglutide 2.4 mg served as a supplementary non-governmental retail scenario.

**Results:**

The pooled file included 76,819 records. An estimated 7.54 million adults had Body Mass Index-defined obesity, 9.04 million met the broad criterion, and 3.67 million met pragmatic priority. At 100% coverage, broad-criterion annual budgets were S/142.95 billion for liraglutide, S/23.65 billion for the IETSI (Institute for Health Technology Assessment and Research)-reported naltrexone/bupropion scenario, and S/26.83 billion for phentermine/topiramate; corresponding pragmatic-priority budgets were S/57.95 billion, S/9.59 billion, and S/10.88 billion. Semaglutide 2.4 mg cost S/16,804.86 per patient in the first year and S/18,135.52 in a maintenance year.

**Conclusion:**

In Peru, a substantial proportion of adults meet survey-observable criteria for treatment with obesity medications. The broad criterion reaches more than nine million adults, whereas pragmatic priority reduces the initial candidate population to nearly four million. Projected budget impact is driven primarily by candidate-population size and annual medication cost. Liraglutide 3.0 mg and semaglutide 2.4 mg represent high-cost non-governmental retail scenarios rather than expected institutional procurement estimates. Extended-release naltrexone/bupropion provides a lower-cost non–GLP-1 comparator,and extended-release phentermine/topiramate was analyzed only as an exploratory supplementary comparator.

## Graphical abstract

**Figure:**
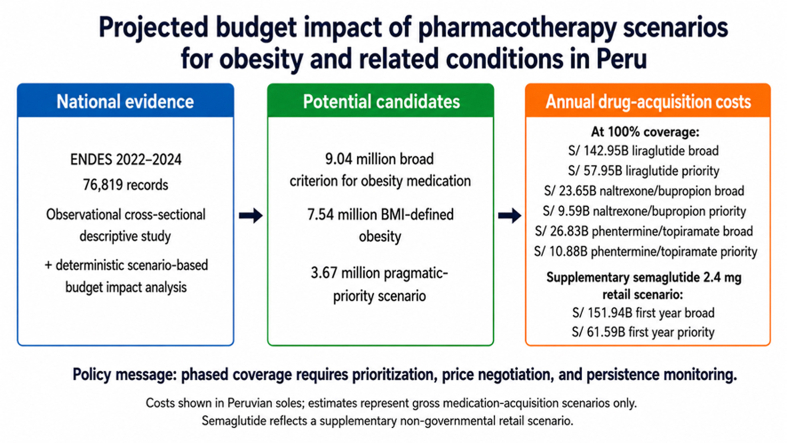

## 1 Introduction

Excess weight and obesity are population-scale health problems that can no longer be treated as isolated risk factors. In 2022, more than one billion people were living with obesity; adult obesity prevalence had more than doubled and obesity among children and adolescents had quadrupled since 1990. Global Burden of Disease (GBD) projections suggest that, if current trajectories persist, nearly 60% of adults will have overweight or obesity by 2050. The clinical relevance of this trend is explained by its association with cardiovascular disease, type 2 diabetes, kidney disease, and other chronic outcomes; in the GBD 2015 analysis, high body mass index was linked to approximately 4 million deaths, predominantly from cardiovascular causes [[1], [2], [3]].

In Latin America, the problem combines nutritional transition, urbanization, social inequality, and limited health-financing capacity for chronic therapies. A Latin American consensus on obesity pharmacotherapy recommended pharmacologic treatment in adults with a body mass index of 30 kg/m^2^ or higher, or 27 kg/m^2^ or higher with at least one comorbidity, always as part of an individualized, multidisciplinary strategy. In Peru, the magnitude of the problem varies according to the anthropometric indicator used: a national systematic review and meta-analysis estimated obesity prevalences of 23.23% by body mass index, 38.90% by waist circumference, and 81.53% by waist-to-height ratio. In addition, an analysis of Peruvian national surveys from 2014 to 2022 estimated that 27.7% of adults met criteria for pharmacologic obesity management and 2.5% for bariatric surgery. These data indicate that the debate is not limited to estimating prevalence, but also to identifying what proportion of the population could enter more intensive therapeutic pathways [[4], [5], [6]].

The clinical context has changed for two reasons. First, obesity is increasingly recognized as a heterogeneous condition in which body mass index, although useful for population surveillance, may underestimate or overestimate adiposity and does not by itself capture individual clinical impairment. Second, modern pharmacotherapy has been incorporated as an adjunct to nutritional, physical, and behavioral management in adults with obesity or overweight with weight-related complications. Semaglutide 2.4 mg and tirzepatide have shown substantial weight reductions in randomized trials, and the SELECT trial showed a reduction in major cardiovascular events with semaglutide in people with overweight or obesity and established cardiovascular disease, without diabetes [[7], [8], [9], [10], [11], [12]].

However, clinical efficacy does not automatically resolve the health-policy question that matters for a middle-income country: how many people would be candidates, under which criteria, how much it would cost to treat them, and which insurance strata would concentrate the budgetary pressure. This distinction is central because modern drugs for obesity and cardiometabolic risk have high prices and substantial international variability; moreover, recent estimates suggest that market prices for several modern diabetes therapies may greatly exceed potential production costs under generic or biosimilar competition. Therefore, before discussing universal coverage or phased implementation, a budget impact analysis is required; this approach is designed to estimate the financial consequences of incorporating health technologies in a defined population [13,14].

Peru does not operate as a single-payer health system. Health financing and service delivery are distributed across the Ministry of Health and regional service networks, the Seguro Integral de Salud (SIS), EsSalud for formal-sector workers and their dependents, smaller public subsystems, and private providers and insurers; a residual population remains uninsured. These arrangements differ in provider networks, formularies, procurement mechanisms, referral pathways, and specialist capacity. Accordingly, ENDES (Encuesta Demográfica y de Salud Familiar or Demographic and Family Health Survey) insurance categories were used to identify where potential budget pressure may concentrate, rather than to assign definitive payer responsibility or assume uniform implementation capacity [15].

Available Peruvian evidence has estimated obesity prevalence, eligibility for obesity management, and diabetes prevalence; however, it has not quantified the adult population potentially eligible for treatment with obesity medications or the projected annual medication-acquisition cost under alternative prioritization, coverage, price, and health-insurance scenarios. This study aimed to estimate, using recent national data, the number and proportion of Peruvian adults meeting survey-observable criteria for treatment with obesity medications and to project annual medication-acquisition costs for liraglutide 3.0 mg as a high-cost non-governmental scenario, extended-release naltrexone/bupropion as the main non–glucagon-like peptide-1 (GLP-1) obesity-medication comparator, extended-release phentermine/topiramate as an exploratory supplementary comparator, and semaglutide 2.4 mg as a supplementary non-governmental retail scenario with separate first-year and maintenance-year costs. Metformin and sodium-glucose cotransporter-2 (SGLT2) inhibitors were analyzed only as secondary cardiometabolic comparators because of their relevance to diabetes-related comorbidity and budgetary scale, not as obesity medications [5,6,14,16].

## 2 Methods

### 2.1 Study design

This was an observational, cross-sectional descriptive study with a deterministic scenario-based budget impact analysis component. The analysis used anonymized secondary microdata from the ENDES corresponding to the 2022, 2023, and 2024 rounds. The epidemiologic component estimated the number and proportion of adults with survey-observable profiles compatible with treatment using obesity medications and with secondary cardiometabolic comparator scenarios; the economic component projected gross annual medication-acquisition requirements under explicit coverage and price-source assumptions. The manuscript was prepared according to the STROBE recommendations for cross-sectional observational studies [17].

The unit of inference was the adult population residing in Peru and represented by the ENDES sampling design during the study period. The study was not designed to estimate causal effects, individual clinical effectiveness, or incremental cost-effectiveness. Its primary estimand was the population size of survey-observable candidates for treatment with obesity medications and the annual gross medication-acquisition budget associated with different prioritization and treatment strategies.

### 2.2 Data source

ENDES is a continuous population-based survey conducted by the National Institute of Statistics and Informatics of Peru. Its objective is to produce updated information on demographic dynamics, maternal and child health, communicable and noncommunicable diseases, and access to diagnostic and treatment services. The target population includes private households and their members; in the health module, one person aged 15 years or older is selected per household for interview and physical measurements. The sampling design is probabilistic, two-stage, stratified, and independent by study domains, with national, urban-rural, natural-region, and departmental inference [18].

We used variables from the health questionnaire, anthropometry, blood pressure, history of diabetes, use of antihypertensive medication, sociodemographic characteristics, and reported insurance. Original data collection was conducted through face-to-face interviews and standardized field procedures. ENDES questionnaires were administered by trained field personnel rather than self-completed. The category “no education” reflects reported formal educational attainment and should not be interpreted as a direct measure of literacy. For this analysis, we used a frozen analytic dataset derived from the 2022–2024 rounds.

### 2.3 Study population

The main population comprised adults aged 18 years or older with valid information for the variables required by each therapeutic scenario. Obesity-medication analyses were defined among adults aged 18 years or older because the main question was the population magnitude of adult obesity potentially amenable to pharmacologic treatment. For scenarios restricted to diabetes, metformin, and sodium–glucose cotransporter 2 inhibitors, we used a subpopulation of adults aged 30 years or older with self-reported diagnosed diabetes. This age restriction was specified to increase specificity toward a phenotype compatible with type 2 diabetes because ENDES does not distinguish type 1 diabetes, type 2 diabetes, gestational diabetes, or other forms of diagnosed hyperglycemia.

We did not perform a global deletion of participants with missing auxiliary variables. Each participant contributed to analyses for which valid information was available; therefore, denominators varied by indicator and scenario. Participants with non-missing clinical measurements but missing pooled survey weights remained in the source file but did not contribute to design-weighted estimates. Accordingly, BODY Mass Index (BMI)-based survey estimates required non-missing BMI, a non-missing pooled survey weight, and complete stratum and primary-sampling-unit information. Conventional codes for nonresponse or invalid measurement were treated as missing. Continuous variables were cleaned using physiologically plausible ranges defined before analysis: systolic blood pressure 70–270 mmHg, diastolic blood pressure 30–150 mmHg, weight 25–300 kg, height 120–220 cm, waist circumference 40–220 cm, and body mass index 10–80 kg/m^2^.

### 2.4 Variables and operational definitions

Body mass index (BMI) was calculated as weight in kilograms divided by height in meters squared. BMI categories were defined using half-open intervals: <18.5, 18.5–<25, 25–<30, 30–<35, 35–<40, and ≥40 kg/m^2^. Obesity was defined as BMI ≥30 kg/m^2^; severe obesity as BMI ≥35 kg/m^2^; and very severe obesity as BMI ≥40 kg/m^2^. Measured hypertension was defined as systolic blood pressure ≥140 mmHg or diastolic blood pressure ≥90 mmHg, a threshold widely used in population epidemiology of hypertension [19]. Probable or treated hypertension combined measured hypertension or self-reported use of antihypertensive medication.

Diagnosed diabetes was defined by self-report of a previous diagnosis of diabetes or high blood sugar by a physician or another health worker. Observable comorbidity for obesity-medication scenarios was defined as diagnosed diabetes or probable/treated hypertension. Reported health insurance was categorized as uninsured, Seguro Integral de Salud, EsSalud, and other insurance. This variable was used as an analytic stratum of potential budgetary pressure, not as definitive administrative attribution of the payer.

Primary eligibility scenarios for obesity medications were defined hierarchically. The obesity scenario included adults with BMI ≥30 kg/m^2^. The broad scenario included adults with BMI ≥30 kg/m^2^, or BMI 27–<30 kg/m^2^ with diagnosed diabetes or probable/treated hypertension. The pragmatic-priority scenario included BMI ≥35 kg/m^2^, or BMI 30–<35 kg/m^2^ with diagnosed diabetes or probable/treated hypertension. Scenarios restricted to severe obesity, very severe obesity, diagnosed diabetes with BMI ≥27 kg/m^2^, and diagnosed diabetes with obesity were also estimated. These populations were used to cost liraglutide, extended-release naltrexone/bupropion, exploratory extended-release phentermine/topiramate, and supplementary semaglutide scenarios. They were interpreted as survey-observable potential candidates, not as individual clinical eligibility.

Secondary cardiometabolic comparator scenarios were restricted to adults aged 30 years or older with diagnosed diabetes. The maximum diabetes scenario included the entire diagnosed-diabetes subpopulation; the main SGLT2-inhibitor comparator scenario used a cardiometabolic risk proxy defined as diagnosed diabetes plus obesity or probable/treated hypertension. Metformin comparator scenarios were restricted to diagnosed diabetes and, secondarily, to diagnosed diabetes with obesity. Dapagliflozin, empagliflozin, and metformin were analyzed as secondary cardiometabolic comparators rather than obesity-medication scenarios.

### 2.5 Price sources and pharmacologic assumptions

The price matrix was built using a prespecified hierarchy. Peruvian normative and institutional sources were prioritized, including the National Single List of Essential Medicines, ministerial complementary lists, health technology assessments from the General Directorate of Medicines, Supplies, and Drugs (DIGEMID, for its Spanish name, Dirección General de Medicamentos, Insumos y Drogas) and the Institute for Health Technology Assessment and Research of EsSalud (IETSI-EsSalud, for its Spanish name, Instituto de Evaluación de Tecnologías en Salud e Investigación de EsSalud), and public FARMAMINSA price lists. Documented retail prices were used only when no verifiable public institutional price was identified and were interpreted as non-governmental scenarios rather than expected public procurement prices.

The National Single List of Essential Medicines approved through Ministerial Resolution 633-2023-MINSA was used as the normative source for essential medicines. In the insulin and other antidiabetic-drug group, metformin 500 mg and 850 mg, glibenclamide, gliclazide, and insulins were identified, but unit prices were not identified in the list annex [20]. For SGLT2 inhibitors, Ministerial Resolution 182-2025-MINSA approved a complementary list for type 2 diabetes mellitus and arterial hypertension to the National Single List of Essential Medicines (PNUME, for its Spanish name, Petitorio Nacional Único de Medicamentos Esenciales), and DIGEMID Short Review 07–2024 agreed to include empagliflozin 10 mg and 25 mg and dapagliflozin 10 mg for type 2 diabetes mellitus in adult patients with established cardiovascular disease, heart failure, or diabetic nephropathy, as well as high cardiovascular risk under specialist indication [21,22].

For metformin, the baseline scenario used 850 mg every 12 h. The unit price of S/0.06 per tablet came from the FARMAMINSA Surco price list dated February 2, 2026; the annual cost was 2 tablets daily for 365 days, equivalent to S/43.80 per patient-year [23]. For SGLT2 inhibitors, the base scenario used dapagliflozin 10 mg daily at S/5.40 per tablet and S/1971.00 per patient-year. Alternatives included empagliflozin 10 mg daily at S/2445.50 per patient-year, reported by IETSI-EsSalud, and empagliflozin 25 mg daily at S/9.40 per tablet and S/3431.00 per patient-year, reported by DIGEMID [22,24].

Liraglutide 3.0 mg daily was modeled as a high-cost non-governmental scenario because its Peruvian technical sheet documents an indication in adults with body mass index (BMI) ≥30 kg/m^2^, or BMI 27–<30 kg/m^2^ with at least one weight-related comorbidity, together with a hypocaloric diet and increased physical activity. Each pen contains 18 mg; at 3.0 mg daily, approximately 60.83 pens are required per patient-year. The annual cost was S/15,810.57, derived from a documented retail price of approximately S/259.90 per pen. Because no consolidated national institutional procurement price for liraglutide 3.0 mg for obesity was identified, this retail value was modeled only as a high-cost non-governmental ceiling scenario [25,26].

Extended-release naltrexone/bupropion served as the main non–GLP-1 obesity-medication comparator. IETSI-EsSalud reported a unit cost of S/1.35 and an annual cost of S/2615.90 without reporting the annualization formula. Accordingly, S/2615.90 served as the reported health technology assessment (HTA) annual-cost scenario, while arithmetic estimates of S/1971.00 for a full maintenance year and S/1914.30 for the first year with labeled dose escalation were analyzed in sensitivity analyses [27,28]. Extended-release phentermine/topiramate was analyzed as an exploratory supplementary comparator at S/2967.45 per patient-year. Because the IETSI assessment did not approve its use as a non-formulary product in EsSalud and raised benefit–risk concerns, this scenario was not interpreted as a coverage or prescribing recommendation [29].

Semaglutide 2.4 mg weekly was modeled as a supplementary high-cost non-governmental retail scenario. The DIGEMID technical sheet confirms that each 3-mL FlexTouch pen contains four 2.4-mg doses. The first-year cost incorporated the labeled 16-week dose-escalation schedule and was S/16,804.86 using Farmacia Universal prices available for all strengths; the maintenance-year cost was S/18,135.52, corresponding to 13 four-dose pens per year. A Farmacenter listing yielded an alternative maintenance-price sensitivity of S/16,744.00. These retail scenarios were not interpreted as institutional procurement prices, coverage recommendations, or cost-effectiveness estimates [[30], [31], [32], [33], [34], [35], [36]].

Selected Peruvian retail listings were identified for tirzepatide strengths and an orlistat product; however, a complete, indication-concordant, and reproducible annual obesity-treatment regimen could not be constructed from verified regulatory and price sources. These products were therefore not costed [[37], [38], [39]].

The reference scenario assumed no new programmatic acquisition of the modeled obesity medications and held existing condition-related care costs constant. Accordingly, projected values represent gross additional medication-acquisition requirements rather than net budget changes after treatment substitution, monitoring, adverse events, or avoided clinical events. Medication-acquisition costs did not include consultations, laboratory tests, administration, monitoring, adverse events, medication-specific longitudinal discontinuation, switching, re-initiation, or indirect costs.

### 2.6 Statistical analysis

All analyses incorporated the complex ENDES sampling design using weights, strata, and primary sampling units. To combine the 2022–2024 rounds, the annual weight was rescaled by dividing it by three so that the pooled file represented an average population for the period rather than an artificial accumulation of three annual populations. Variance was estimated by Taylor linearization, using QHCLUSTER as the primary sampling unit, HV022 as the stratum, and centering for strata with a single primary sampling unit. The survey design was specified on the full file, and analytic-population estimates were obtained using subpopulations rather than prior trimming of the database, preserving the correct variance structure under the survey design.

Continuous variables were summarized using weighted means and 95% confidence intervals; categorical variables were summarized using weighted proportions and 95% confidence intervals. For each therapeutic scenario, unweighted counts, weighted proportions, weighted population totals, and 95% confidence intervals were estimated. Results were reported with scenario-specific denominators because criteria based on body mass index require valid anthropometry, whereas diagnosed-diabetes scenarios should not lose observations due to missing anthropometry when anthropometry is not part of the criterion.

Annual budget impact was calculated using the formula: projected annual cost equals the population number of candidates multiplied by annual cost per treated patient, programmatic coverage, and annual persistence. The main cost estimates used programmatic coverage levels of 20%, 50%, 80%, and 100%, while maintaining annual persistence at 100% to represent a direct budgetary ceiling. The structural sensitivity analysis of the high-cost non-governmental liraglutide 3.0 mg pragmatic-priority scenario used 50% coverage and 80% persistence as the base case and varied the scenario population size, programmatic coverage, annual price per patient, annual persistence, and comorbidity definition.

Adjusted regression models were not used as the main analysis. This decision was deliberate: the objective of the study was to quantify candidate populations and absolute costs for health planning, not to estimate etiologic associations or independent effects of an exposure. In this context, multivariable adjustment would have shifted the estimand toward a causal question not posed and less useful for budget impact. Annual estimates for 2022, 2023, and 2024 were calculated as an internal stability analysis, not as a formal trend analysis.

Structural sensitivity analyses assessed alternative candidate definitions, programmatic coverage, annual persistence, and price assumptions. For the broad and pragmatic-priority obesity-medication scenarios, probable/treated hypertension was replaced by measured hypertension only, and the overweight comorbidity criterion was restricted to diagnosed diabetes. Adiposity definitions based on elevated waist circumference and waist-to-height ratio ≥0.50 were estimated separately but were not used as principal pharmacologic criteria. Additional pharmacologic cost-source sensitivity analyses evaluated arithmetic naltrexone/bupropion costs and alternative semaglutide maintenance pricing. Annual estimates for 2022, 2023, and 2024 were treated as internal stability analyses rather than formal trend analyses. Main processing was performed in Stata 17.0; final tables and figures were verified against the analytic outputs.

### 2.7 Ethical considerations

This study used publicly available, de-identified secondary data from ENDES. Because the analysis involved no direct participant contact and no access to identifiable private information, formal review by the UNTRM institutional ethics committee and additional informed consent for this secondary analysis were not required. Original ENDES data collection was conducted by INEI under its institutional procedures for informed consent, confidentiality, and data protection [18].

## 3 Results

### 3.1 Participant selection and general characteristics

The pooled ENDES 2022–2024 analytic file included 76,819 records, including 69,414 adults aged 18 years or older. A total of 69,019 adults had a non-missing pooled survey weight and complete stratum and primary-sampling-unit information and contributed to design-weighted adult estimates. BMI was non-missing in 68,358 adults; 68,043 had both non-missing BMI and a non-missing pooled survey weight and therefore formed the denominator for design-weighted BMI-based estimates. The diabetes-related source branch included 49,363 adults aged 30 years or older. Of these, 49,053 had a non-missing pooled survey weight and 49,011 had both a non-missing pooled survey weight and non-missing diabetes status. Diagnosed diabetes was reported by 2599 adults, of whom 2577 had a non-missing pooled survey weight and contributed to weighted estimates. The parallel denominators are shown in Fig. S1, and missingness is detailed in Table S3.

Adult population characteristics are presented in Table 1. The weighted mean age was 43.2 years (95% CI: 43.0–43.4), 51.0% were women, and 83.5% resided in urban areas. Reported coverage corresponded mainly to SIS (55.3%) and EsSalud (25.4%), whereas 14.7% reported no health insurance. Mean BMI was 27.7 kg/m^2^ (95% CI: 27.6–27.8). Overall, 69.3% had overweight or obesity, 28.2% had obesity based on BMI ≥30 kg/m^2^, 73.9% had elevated waist circumference, and 87.3% had a waist-to-height ratio ≥0.50. Among adults with observed BMI and pooled survey weight, 27,759/68,043 were classified as BMI 25–<30 kg/m^2^, representing 41.2% after survey weighting. Diagnosed diabetes was reported by 5.8% of adults and probable or treated hypertension by 19.5%.

**Table 1:** Characteristics of the pooled adult ENDES sample, 2022–2024.

Characteristic	n	Weighted estimate (95% CI)
Continuous variables		
Age, years	69,019	43.2 (43.0–43.4)
BMI, kg/m^2^	68,043	27.7 (27.6–27.8)
Waist circumference, cm	67,227	93.1 (93.0–93.3)
Systolic blood pressure, mmHg	68,579	120.1 (119.9–120.4)
Diastolic blood pressure, mmHg	68,591	75.5 (75.3–75.6)
Sociodemographic variables		
Sex		
Male	30,190/69,019	49.0% (48.3–49.6%)
Female	38,829/69,019	51.0% (50.4–51.7%)
Area of residence		
Urban	45,576/69,019	83.5% (82.9–84.2%)
Rural	23,443/69,019	16.5% (15.8–17.1%)
Natural region		
Metropolitan Lima	8377/69,019	39.3% (38.0–40.5%)
Rest of the coast	19,928/69,019	25.7% (24.6–26.7%)
Highlands	23,978/69,019	22.8% (21.8–23.8%)
Jungle	16,736/69,019	12.3% (11.6–12.9%)
Wealth quintile		
Q1 - poorest	21,560/69,019	17.0% (16.3–17.6%)
Q2 - poor	17,193/69,019	19.4% (18.8–20.0%)
Q3 - middle	13,070/69,019	21.2% (20.6–21.7%)
Q4 - rich	10,052/69,019	21.5% (20.8–22.1%)
Q5 - richest	7144/69,019	21.0% (20.2–21.8%)
Educational level		
No education	139/66,586	0.2% (0.1–0.2%)
Primary	14,861/66,586	18.5% (18.0–19.1%)
Secondary	29,969/66,586	44.6% (43.9–45.3%)
Higher education	21,617/66,586	36.7% (36.0–37.5%)
Health insurance		
Uninsured	8361/69,019	14.7% (14.2–15.1%)
SIS	45,644/69,019	55.3% (54.5–56.0%)
EsSalud	13,281/69,019	25.4% (24.7–26.0%)
Other	1733/69,019	4.7% (4.4–5.1%)
Adiposity and cardiometabolic profile		
BMI category		
BMI <18.5	695/68,043	1.1% (0.9–1.2%)
BMI 18.5–<25	22,063/68,043	29.6% (29.0–30.1%)
BMI 25–<30	27,759/68,043	41.2% (40.6–41.8%)
BMI 30–<35	13,069/68,043	20.7% (20.2–21.3%)
BMI 35–<40	3397/68,043	5.5% (5.2–5.8%)
BMI ≥40	1060/68,043	2.0% (1.8–2.1%)
Obesity, BMI ≥30 kg/m^2^	17,526/68,043	28.2% (27.5–28.8%)
Overweight/obesity, BMI ≥25 kg/m^2^	45,285/68,043	69.3% (68.8–69.9%)
Elevated waist circumference	48,205/67,227	73.9% (73.4–74.5%)
Waist-to-height ratio ≥0.50	58,657/67,208	87.3% (86.9–87.8%)
Diagnosed diabetes	2735/68,959	5.8% (5.4–6.1%)
Measured elevated BP, ≥140/90 mmHg	7897/68,579	15.5% (15.0–16.0%)
Probable hypertension: measurement or treatment	9861/68,604	19.5% (18.9–20.0%)
Observable comorbidity: diabetes or hypertension	11,485/68,568	22.5% (21.9–23.1%)

Continuous variables are presented as weighted means (95% CIs), and categorical variables as weighted proportions (95% CIs). Counts are unweighted and reflect the available denominator for each variable. BMI = body mass index; BP = blood pressure; CI = confidence interval; ENDES = Peruvian Demographic and Family Health Survey; SIS = Seguro Integral de Salud. Weighted estimates represent the adult population under the ENDES sampling design. The category “no education” indicates no formal schooling and does not directly measure literacy. Socioeconomic variables were descriptive and were not used to define medication eligibility or calculate budget impact.

### 3.2 Potential candidates for obesity medications and secondary comparator scenarios

Table 2 summarizes the analytic scenarios. The broad obesity-medication criterion, defined as BMI ≥30 kg/m^2^ or BMI 27–<30 kg/m^2^ with diagnosed diabetes or probable/treated hypertension, identified 20,300 candidates among 68,030 adults with the required data, equivalent to a weighted 33.8% and 9.04 million adults. BMI-defined obesity identified 7.54 million adults. The pragmatic-priority scenario, defined as BMI ≥35 kg/m^2^ or BMI 30–<35 kg/m^2^ with diabetes or probable/treated hypertension, identified 3.67 million adults. Severe obesity and BMI ≥40 kg/m^2^ identified 1.99 million and 0.52 million adults, respectively.

**Table 2:** Survey-observable therapeutic-scenario populations.

Therapeutic scenario	Target population	n/N	Weighted % (95% CI)	Estimated population, millions (95% CI)
pharmacotherapy for obesity: BMI ≥30 kg/m^2^	Adults ≥18 years	17,526/68,043	28.2% (27.5–28.8%)	7.54 (7.28–7.79)
pharmacotherapy for obesity: broad criterion	Adults ≥18 years	20,300/68,030	33.8% (33.1–34.5%)	9.04 (8.75–9.33)
pharmacotherapy for obesity: pragmatic priority	Adults ≥18 years	7509/68,034	13.7% (13.2–14.2%)	3.67 (3.50–3.83)
pharmacotherapy for obesity: severe obesity	Adults ≥18 years	4457/68,043	7.4% (7.1–7.8%)	1.99 (1.88–2.10)
pharmacotherapy for obesity: very severe obesity	Adults ≥18 years	1060/68,043	2.0% (1.8–2.1%)	0.52 (0.47–0.57)
pharmacotherapy for obesity: diagnosed diabetes + BMI ≥27	Adults ≥30 years	1598/48,310	4.9% (4.6–5.3%)	0.96 (0.88–1.04)
pharmacotherapy for obesity: diagnosed diabetes + obesity	Adults ≥30 years	981/48,310	3.1% (2.8–3.4%)	0.60 (0.53–0.66)
Secondary cardiometabolic comparator: metformin, diagnosed diabetes	Adults ≥30 years	2577/49,011	7.6% (7.2–8.1%)	1.51 (1.41–1.61)
Secondary cardiometabolic comparator: metformin, diagnosed diabetes + obesity	Adults ≥30 years	981/48,310	3.1% (2.8–3.4%)	0.60 (0.53–0.66)
Secondary cardiometabolic comparator: SGLT2 inhibitor, diagnosed diabetes	Adults ≥30 years	2577/49,011	7.6% (7.2–8.1%)	1.51 (1.41–1.61)
Secondary cardiometabolic comparator: SGLT2 inhibitor, diabetes + risk proxy	Adults ≥30 years	1618/48,975	5.1% (4.7–5.5%)	1.01 (0.93–1.10)

Estimates represent potential candidates observable in ENDES and do not correspond to individual clinical eligibility. BMI = body mass index; CI = confidence interval; ENDES = Peruvian Demographic and Family Health Survey; SGLT2 = sodium–glucose cotransporter 2.

In secondary cardiometabolic comparator scenarios, diagnosed diabetes among adults aged 30 years or older corresponded to 1.51 million people, used as the metformin scenario and as the upper bound for SGLT2 inhibitors in diabetes. The SGLT2-inhibitor scenario with a risk proxy, defined as diagnosed diabetes plus obesity or probable/treated hypertension, identified 1.01 million people. The obesity-medication scenario restricted to diabetes with obesity identified 0.60 million people.

### 3.3 Unit prices and annual cost per treated patient

Table 3 presents the annual per-patient costs used in the model. Liraglutide 3.0 mg was modeled as a high-cost non-governmental scenario at S/15,810.57 per patient-year. Extended-release naltrexone/bupropion served as the main non–GLP-1 obesity-medication comparator at the IETSI-reported annual cost of S/2615.90; arithmetic estimates of S/1971.00 for a maintenance year and S/1914.30 for the first year with dose escalation were examined in sensitivity analyses [27,28]. Extended-release phentermine/topiramate was modeled as an exploratory supplementary comparator at S/2967.45 per patient-year [29]. Semaglutide 2.4 mg weekly was modeled as a supplementary non-governmental retail scenario, with a first-year cost of S/16,804.86 and a maintenance-year cost of S/18,135.52; an alternative retail listing yielded a maintenance-year sensitivity of S/16,744.00 [[30], [31], [32], [33], [34], [35], [36]]. For the secondary cardiometabolic comparators, metformin 850 mg every 12 h had an annual cost of S/43.80 per patient; dapagliflozin 10 mg daily was the base SGLT2-inhibitor regimen at S/1971.00 per patient-year, and empagliflozin 10 mg and 25 mg were alternative regimens at S/2445.50 and S/3431.00 per patient-year, respectively [[22], [23], [24]].

**Table 3:** Medication costs and roles in the deterministic acquisition model.

Medication/regimen	Associated scenario	Annual cost per patient	Price source	Use in the model
Liraglutide 3.0 mg daily	pharmacotherapy for obesity	S/15,810.57	Documented Peruvian retail source [25,26]	Primary high-cost non-governmental ceiling scenario
Semaglutide 2.4 mg once weekly	Supplementary non-governmental retail scenario	S/16,804.86 first year; S/18,135.52 maintenance	Farmacia Universal retail listings and DIGEMID technical sheet [[30], [31], [32], [33], [34], [35]]	Supplementary retail scenario; not an institutional acquisition estimate
Naltrexone/bupropion ER	Non-GLP-1 pharmacotherapy for obesity	S/2615.90	IETSI-EsSalud HTA [27]	Main non-GLP-1 obesity-medication comparator
Phentermine/topiramate ER	Exploratory non-GLP-1 pharmacotherapy for obesity	S/2967.45	IETSI-EsSalud HTA [29]	Exploratory supplementary comparator; not interpreted as coverage recommendation
Dapagliflozin 10 mg daily	SGLT2 inhibitor, diabetes + risk proxy	S/1971.00	DIGEMID HTA [22]	Secondary SGLT2-inhibitor comparator base
Empagliflozin 10 mg daily	SGLT2 inhibitor, diabetes + risk proxy	S/2445.50	IETSI-EsSalud HTA [24]	Secondary SGLT2-inhibitor alternative
Empagliflozin 25 mg daily	SGLT2 inhibitor, diabetes + risk proxy	S/3431.00	DIGEMID HTA [22]	Secondary SGLT2-inhibitor alternative
Metformin 850 mg every 12 h	Diagnosed diabetes ≥30 years	S/43.80	FARMAMINSA public price list [23]	Secondary low-cost cardiometabolic comparator

Semaglutide first-year cost incorporates the labeled 16-week dose-escalation schedule and 36 subsequent weeks at 2.4 mg. Maintenance assumes 13 four-dose pens per year. For extended-release naltrexone/bupropion, S/2615.90 is the annual cost reported by IETSI-EsSalud; arithmetic maintenance-year and first-year dose-escalation estimates are presented in Table S10. ER = extended release; GLP-1 = glucagon-like peptide-1; HTA = health technology assessment; IETSI = Instituto de Evaluación de Tecnologías en Salud e Investigación; SGLT2 = sodium–glucose cotransporter 2. Retail prices represent non-governmental scenarios rather than institutional acquisition prices. Farmacenter was used only as an alternative semaglutide maintenance-price sensitivity.

### 3.4 Projected budget impact

**Fig. 1:**
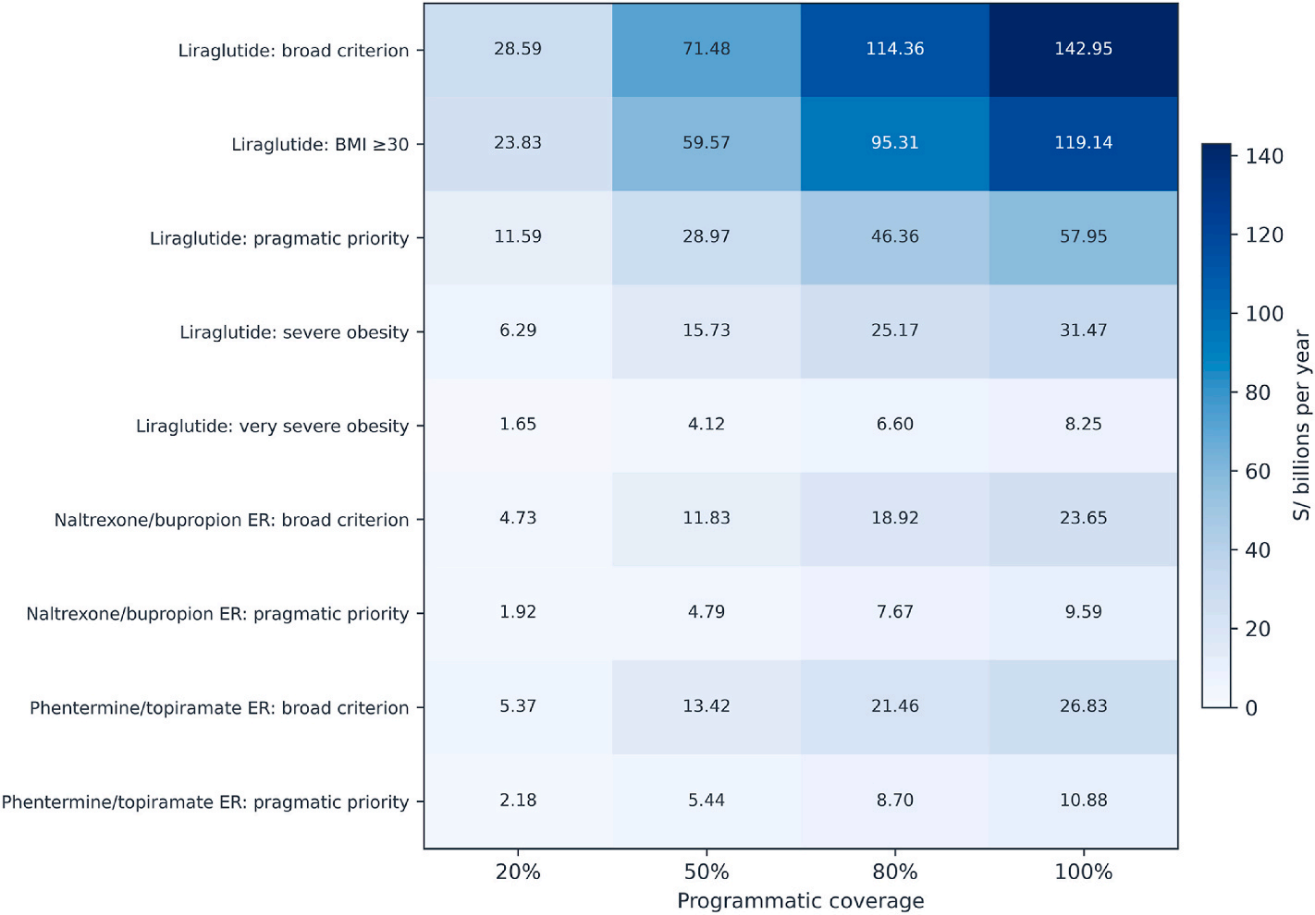
Projected annual medication-acquisition budget under the principal obesity-medication scenarios by programmatic coverage. Values are expressed as S/billions per year and assume 100% annual persistence. Liraglutide 3.0 mg was modeled as a high-cost non-governmental scenario based on documented retail pricing. Extended-release naltrexone/bupropion served as the main non–GLP-1 obesity-medication comparator, and extended-release phentermine/topiramate was analyzed as an exploratory supplementary comparator. BMI = body mass index; ER = extended release.

Fig. 1 presents the annual medication-acquisition budget for the principal obesity-medication scenarios by programmatic coverage. Under the high-cost non-governmental liraglutide 3.0 mg scenario, 100% coverage of the broad criterion reached S/142.95 billion annually, whereas pragmatic priority reached S/57.95 billion. Under extended-release naltrexone/bupropion, the corresponding costs were S/23.65 billion and S/9.59 billion. Under exploratory extended-release phentermine/topiramate, they were S/26.83 billion and S/10.88 billion. These estimates show that projected budget impact is driven jointly by candidate-population size and annual medication cost per treated patient.

For semaglutide 2.4 mg, the first-year budget at 100% coverage was S/151.94 billion under the broad criterion and S/61.59 billion under pragmatic priority. The corresponding maintenance-year budgets were S/163.97 billion and S/66.47 billion. These values represent gross non-governmental retail acquisition scenarios and should not be interpreted as public procurement estimates.

Using arithmetic cost alternatives for extended-release naltrexone/bupropion, the broad-criterion budget at 100% coverage decreased from S/23.65 billion under the reported HTA annual-cost scenario to S/17.82 billion for a maintenance year and S/17.31 billion for the first year with dose escalation. Under pragmatic priority, the corresponding budgets were S/9.59 billion, S/7.22 billion, and S/7.02 billion.

Secondary cardiometabolic comparator analyses are presented separately. For the SGLT2-inhibitor scenario in diabetes with a risk proxy, the annual cost at 100% coverage was S/2.00 billion with dapagliflozin, S/2.48 billion with empagliflozin 10 mg, and S/3.48 billion with empagliflozin 25 mg. For metformin in adults aged 30 years or older with diagnosed diabetes, the annual cost at 100% coverage was S/0.07 billion. These scenarios were analyzed as fiscal comparators and should be interpreted separately from obesity-medication scenarios.

### 3.5 Distribution by reported health insurance

**Fig. 2:**
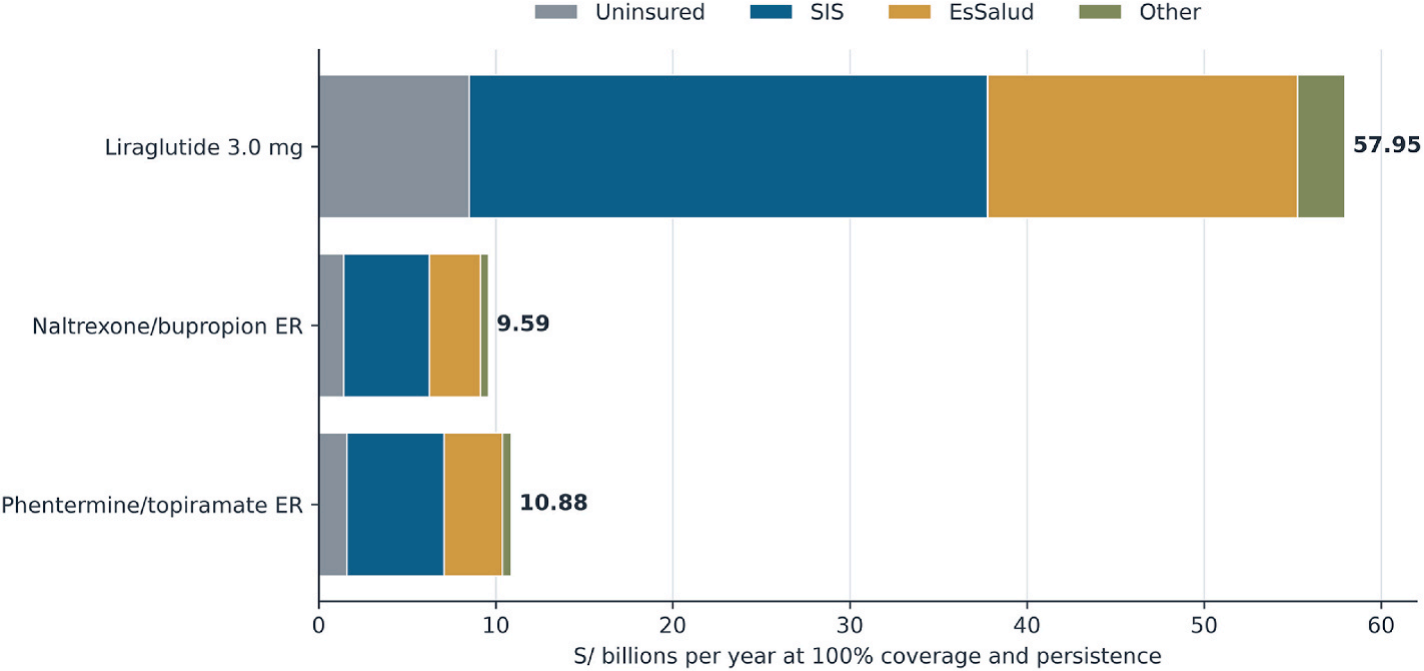
Projected annual medication-acquisition budget of the pragmatic-priority obesity-medication scenario by reported insurance and medication, assuming 100% coverage and 100% persistence. Reported insurance is interpreted as an analytic stratum and not as definitive administrative attribution of the payer. ER = extended release; SIS = Seguro Integral de Salud.

The distribution of candidates by insurance is presented in Table S4, and the pragmatic-priority obesity-medication budget by reported insurance is summarized in Fig. 2. Candidates were distributed mainly in SIS (1.85 million) and EsSalud (1.11 million), followed by uninsured individuals (0.54 million) and other insurance (0.17 million). Under 100% coverage and the liraglutide annual cost used in the high-cost non-governmental scenario, the corresponding budgets were S/29.26 billion in SIS, S/17.50 billion in EsSalud, S/8.51 billion among uninsured individuals, and S/2.68 billion in other insurance.

Using the same pragmatic-priority candidate distribution, extended-release naltrexone/bupropion produced annual costs of S/4.84 billion in SIS, S/2.89 billion in EsSalud, S/1.41 billion among uninsured individuals, and S/0.44 billion in other insurance. Exploratory extended-release phentermine/topiramate produced S/5.49 billion, S/3.28 billion, S/1.60 billion, and S/0.50 billion, respectively. Secondary cardiometabolic comparator estimates by insurance are reported in the supplementary material and should be interpreted separately from obesity-medication scenarios.

### 3.6 Structural sensitivity and internal stability

**Fig. 3:**
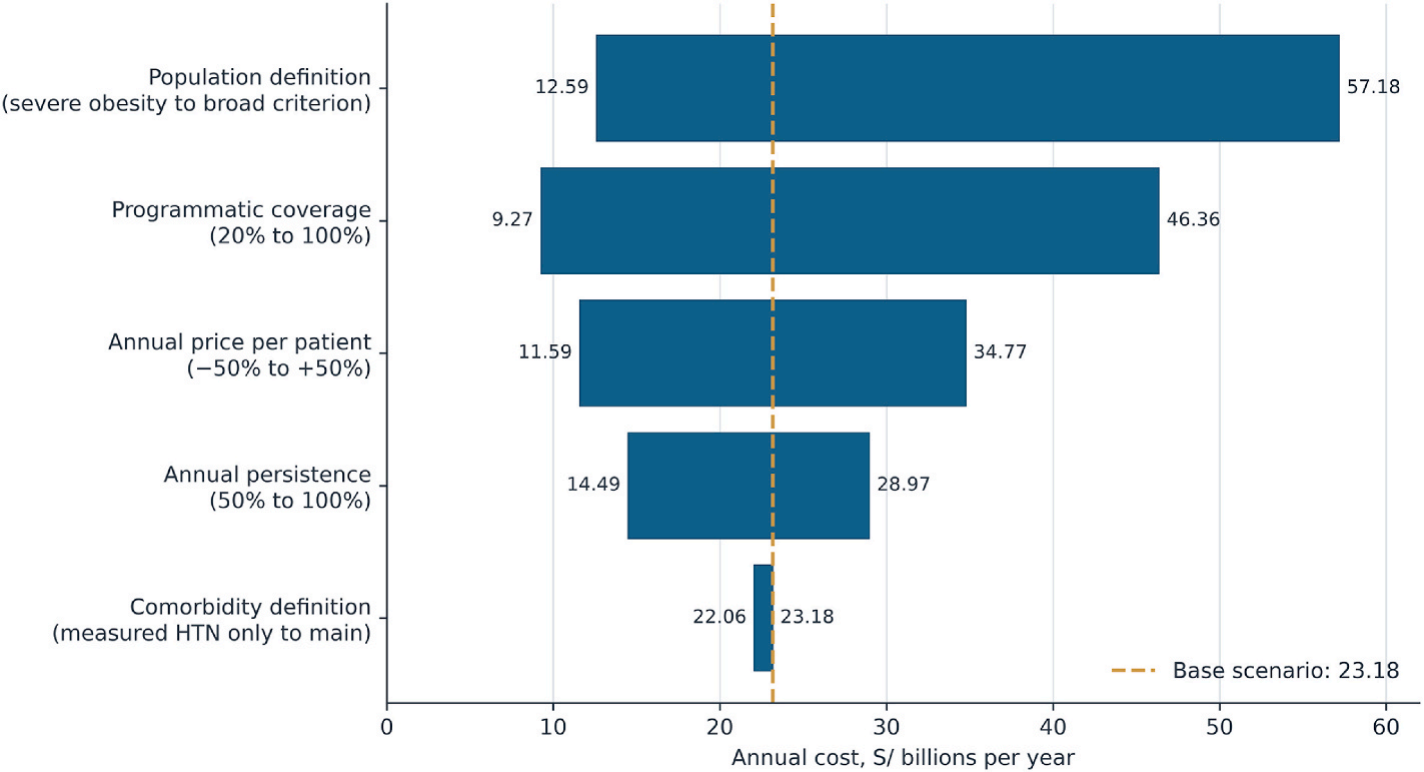
Structural sensitivity of the annual liraglutide 3.0 mg acquisition budget under the pragmatic-priority scenario. The base case assumed 50% programmatic coverage, 80% annual persistence, and S/15,810.57 per patient-year, yielding S/23.18 billion annually. Bars show the range generated by the specified changes in population definition, coverage, annual medication cost, persistence, and comorbidity definition. HTN = hypertension.

Fig. 3 presents the structural sensitivity of the high-cost non-governmental liraglutide 3.0 mg pragmatic-priority scenario. With 50% coverage, 80% annual persistence, and S/15,810.57 per patient-year, the base annual cost was S/23.18 billion. Expansion or restriction of the population definition produced the widest variation range, while programmatic coverage and annual price per patient also substantially modified projected costs. Annual persistence and a stricter hypertension definition produced narrower ranges under the evaluated values.

Definition sensitivity analyses are presented in Table S6. When measured hypertension alone was used, the broad obesity-medication scenario decreased from 9.04 to 8.85 million candidates and pragmatic priority from 3.67 to 3.49 million. When the overweight comorbidity criterion was restricted to diagnosed diabetes, the broad scenario decreased to 7.91 million. Criteria based on abdominal adiposity expanded the estimated burden to 19.61 million with elevated waist circumference and 23.17 million with waist-to-height ratio ≥0.50; these were analyzed as adiposity-burden measures rather than principal pharmacologic criteria.

Annual stability was assessed for 2022, 2023, and 2024 in Table S5 and Fig. S3. BMI-defined obesity ranged from 27.6% to 28.5%, the broad obesity-medication criterion from 33.2% to 34.2%, pragmatic priority from 12.8% to 14.4%, the SGLT2-inhibitor risk-proxy comparator from 4.9% to 5.2%, and the metformin comparator from 7.6% to 7.7%.

## 4 Discussion

### 4.1 Principal findings

This national study estimated that the Peruvian adult population meeting survey-observable criteria for treatment with obesity medications is large and highly sensitive to the prioritization rule. The broad criterion identified 9.04 million adults, BMI-defined obesity identified 7.54 million, and pragmatic priority reduced the initial candidate population to 3.67 million. Liraglutide 3.0 mg generated the highest principal acquisition budgets, followed by the supplementary semaglutide retail scenario. Extended-release naltrexone/bupropion produced lower but still substantial budgets, and extended-release phentermine/topiramate was analyzed only as an exploratory comparator. Secondary cardiometabolic analyses illustrated the affordability gradient across chronic metabolic therapies but were not interpreted as obesity-medication scenarios.

### 4.2 Comparison with other studies

Our results are consistent with global and Peruvian evidence on the growth of obesity, but add a fiscal dimension that was not available in previous estimates. The weighted prevalence of obesity by BMI in adults was 28.2%, a value compatible with the Peruvian review that estimated 23.23% by BMI, although lower than estimates based on waist circumference or waist-to-height ratio. Likewise, the broad pharmacotherapy for obesity criterion reached 33.8%, higher than the 27.7% previously reported for pharmacologic eligibility in Peruvian national surveys from 2014 to 2022; this difference is explained by temporal differences, comorbidity criteria, and the use of a recent 2022–2024 pool. This comparison suggests that the potential need for pharmacotherapy for obesity is neither marginal nor derived from an isolated threshold, but rather an expected consequence of the population burden of excess weight and comorbidity in the country [1,2,5,6].

The contrast between broad and prioritized scenarios aligns with contemporary discussions of clinical obesity. Guidelines recommend pharmacotherapy in adults with BMI ≥30 kg/m^2^ or BMI ≥27 kg/m^2^ with comorbidity, but that rule does not imply immediate universal coverage. Proposed diagnostic criteria for clinical obesity emphasize that BMI is useful for surveillance and initial eligibility but is insufficient to define organ damage, disability, or individual therapeutic priority. Our pragmatic-priority scenario converts classic eligibility rules into an implementation strategy more compatible with budget constraints. Randomized trials and cardiovascular-outcomes evidence support the clinical relevance of modern obesity medications but do not replace a national affordability analysis [4,[7], [8], [9], [10], [11], [12]].

Direct head-to-head trials have shown greater mean weight reduction with semaglutide 2.4 mg than with liraglutide 3.0 mg and with tirzepatide than with semaglutide. In STEP 8, mean body-weight change at 68 weeks was −15.8% with semaglutide and −6.4% with liraglutide [40]. In SURMOUNT-5, least-squares mean body-weight change at 72 weeks was −20.2% with tirzepatide and −13.7% with semaglutide [41]. However, the present study models medication-acquisition costs rather than comparative clinical effectiveness, health outcomes, cost per responder, or cost-effectiveness. Differences in projected acquisition budgets should therefore not be interpreted as rankings of therapeutic efficacy or economic value.

The magnitude of projected costs is consistent with international economic studies identifying medication price and candidate-population size as major determinants of affordability. In Canada, a cost-effectiveness model of semaglutide in people with obesity and cardiovascular disease found strong price dependence, while a United States analysis projected high fiscal costs from expanded Medicare coverage of GLP-1 receptor agonists even after future savings. This study did not estimate QALYs or savings from prevented complications. No medication-specific effects on clinical events, including major adverse cardiovascular events, were incorporated into this acquisition-only model; clinical-outcome and cost-effectiveness comparisons were outside the estimand [14,42,43].

The comparison across obesity-medication and secondary cardiometabolic scenarios shows a wide cost gradient. Liraglutide 3.0 mg and semaglutide 2.4 mg concentrated fiscal pressure because high annual prices were applied to large candidate populations. Extended-release naltrexone/bupropion generated lower but still substantial projected costs, while exploratory extended-release phentermine/topiramate occupied an intermediate non–GLP-1 range and should be interpreted cautiously because the Peruvian HTA did not approve it for EsSalud coverage. Metformin generated a small budget relative to modern therapies, while SGLT2 inhibitors produced an intermediate cost relevant to diabetes and cardiorenal risk rather than isolated obesity. This pattern is consistent with evidence on the gap between market and cost-based prices and with Peruvian studies of chronic-medicine supply [13,[20], [21], [22], [23], [24], [25], [26], [27],^29^,^44^,^45^].

### 4.3 Implications for public and international health

The first implication is that policy for obesity medications should not be framed as a binary choice between no coverage and universal coverage. The broad criterion generated budget pressure far above prioritized scenarios, whereas prioritization by severe obesity or obesity with comorbidity reduced the initial population without abandoning the clinical rationale. For a fragmented system such as Peru's, the same obesity medication may be clinically valuable yet fiscally unviable without an explicit prioritization rule, price negotiation, and persistence monitoring [14,15,21,22,[42], [43], [44]].

The second implication is international. Many low- and middle-income countries face rising obesity, cardiometabolic transition, constrained budgets, and pressure to incorporate modern obesity therapies. This study provides a reproducible sequence for estimating budget impact before coverage: define survey-observable candidates, distinguish obesity-medication scenarios from secondary cardiometabolic comparators, and cross population, price, coverage, and persistence. This avoids extrapolating trial efficacy directly into mass-purchasing decisions [2,[10], [11], [12], [13], [14],^44^].

The third implication concerns equity. The concentration of candidates in SIS and EsSalud implies direct consequences for public financing and social security, while uninsured individuals represent a substantial group whose inclusion may overstate the immediate responsibility of any specific payer. Reported insurance should therefore be interpreted as a stratum of potential budget pressure rather than a definitive administrative allocation [15,18,[20], [21], [22],^45^].

Future decisions on coverage of obesity medications in Peru should follow a staged implementation and evaluation framework. The first stage should prioritize severe obesity and obesity with cardiometabolic comorbidity, together with price negotiation, supply assessment, and prospective patient registration. The second stage should monitor uptake among eligible patients; persistence and discontinuation at 3, 6, and 12 months; weight change; available glycemic and blood-pressure indicators; serious adverse events; equity of access by insurer and region; supply continuity; and actual acquisition and monitoring costs. The third stage should link clinical and administrative data to compare observed expenditure with budget projections and to estimate avoided complications and incremental cost-effectiveness before eligibility is expanded.

## 5 Limitations

This study has several epidemiologic limitations. Its cross-sectional design identifies survey-observable candidates rather than confirming individual clinical eligibility or treatment indication. Diagnosed diabetes, antihypertensive treatment, and insurance were self-reported and may be affected by recall or classification error; anthropometry and blood pressure, however, were measured using standardized ENDES procedures. ENDES does not distinguish diabetes type, and restricting diabetes-related analyses to adults aged 30 years or older improves but does not eliminate this uncertainty. The survey also lacks variables required for individual prescribing decisions, including HbA1c, renal function, albuminuria, lipid profiles, established cardiovascular disease, heart failure, contraindications, patient preferences, previous response to structured lifestyle treatment, psychiatric or seizure history, reproductive safety considerations, and detailed medication history. Reported insurance indicates where potential budget pressure may concentrate but does not identify the actual payer, formulary, procurement channel, referral pathway, or implementation capacity.

The economic results are gross medication-acquisition scenarios rather than payer-specific net budget impact or cost-effectiveness estimates. Retail prices are not institutional acquisition prices, and the semaglutide scenario distinguishes first-year dose escalation from maintenance-year dosing. The IETSI-reported annual naltrexone/bupropion cost did not reconcile with the stated unit price and labeled dosing; the reported HTA value served as the main scenario and arithmetic alternatives were evaluated in sensitivity analyses. Tirzepatide and orlistat were not costed because a complete indication-concordant annual regimen was not established from the verified sources [[37], [38], [39]]. The reference scenario excludes treatment displacement, the costs of untreated obesity, monitoring, adverse events, avoided complications, future health-care savings, indirect costs, and clinical outcomes. Medication persistence was represented through scenario multipliers, but medication-specific longitudinal discontinuation, switching, and re-initiation were not modeled. The estimates should therefore be interpreted as gross acquisition requirements, not total cost of care, net budget impact, or cost-effectiveness.

## 6 Conclusion

In Peru, a substantial proportion of adults meet survey-observable criteria for treatment with obesity medications. The broad criterion reaches more than nine million adults, whereas pragmatic priority reduces the initial candidate population to nearly four million. Projected budget impact is driven primarily by candidate-population size and annual medication cost. Liraglutide 3.0 mg and semaglutide 2.4 mg represent high-cost non-governmental retail scenarios rather than expected institutional procurement estimates. Extended-release naltrexone/bupropion provides a lower-cost non–GLP-1 comparator, and extended-release phentermine/topiramate was analyzed only as an exploratory supplementary comparator.

These findings support a staged implementation strategy based on obesity severity, cardiometabolic comorbidity, explicit price negotiation, and monitoring of persistence, supply, and actual institutional acquisition costs. Expansion toward broader eligibility should be considered only after prospective evaluation of uptake, discontinuation, safety, clinical outcomes, avoided complications, and total budget impact.

**Key clinical takeaway messages**

• National implementation should begin with prioritized groups defined by obesity severity, comorbidity, and cardiometabolic risk rather than immediate universal coverage.
• Medication price and candidate-population size are the main drivers of affordability; price negotiation is essential before broad public financing of high-cost obesity medications.
• Coverage expansion should be paired with monitoring of persistence, supply, clinical outcomes, and institutional prices so budget impact can be updated with real-world data.

## Author contribution

**Victor J. Vera Ponce:** Conceptualization, methodology, formal analysis, investigation, data curation, visualization, project administration, validation, writing - original draft, and writing - review and editing.

## Ethical adherence and ethical review

This study used publicly available, de-identified secondary ENDES data. Because the analysis involved no direct participant contact and no access to identifiable private information, formal review by the UNTRM institutional ethics committee and additional informed consent for this secondary analysis were not required. Original ENDES data collection followed INEI procedures for informed consent, confidentiality, and data protection.

## Data availability

ENDES microdata is publicly available from the Instituto Nacional de Estadistica e Informatica (INEI), Peru. Analytical materials supporting this budget impact analysis are available from the corresponding author on reasonable request.

## Clinical trial registration

Not applicable. This was an observational secondary analysis and not a prospective clinical trial.

## Declaration of artificial intelligence

The authors declare that generative artificial intelligence (AI) and large language models (LLM) were not used in the conception, data analysis, or initial drafting of this manuscript.

## Funding

This study was self-funded and received no specific grant from any funding agency in the public, commercial, or not-for-profit sectors.

## Declaration of competing interests

The author declares no competing interests.
