# Supplementary material for: Projected budget impact of pharmacotherapy scenarios for obesity and related conditions in Peru: A national survey analysis of 76,819 participants
Source: Obes Pillars. 2026 Jul 25;19:100308. doi: 10.1016/j.obpill.2026.100308 (PMC13446298; doi:10.1016/j.obpill.2026.100308)
Supplement: Supplementary file 1 [file mmc1.docx]

**SUPPLEMENTARY MATERIAL**

Table S1. Updated STROBE checklist.

| **Item No.** | **Recommendation** | **Location in the manuscript** |
| --- | --- | --- |
| TITLE AND ABSTRACT | TITLE AND ABSTRACT | TITLE AND ABSTRACT |
| 1 | (a) Indicate the study design with a commonly used term in the title or the abstract. | Title: "Projected Budget Impact of Pharmacotherapy Scenarios for Obesity and Related Conditions in Peru: A National Survey Analysis of 76,819 Participants". Abstract Methods: "This was an observational, cross-sectional descriptive study using ENDES 2022–2024 data, with a deterministic scenario-based budget impact analysis component." |
|  | (b) Provide in the abstract an informative and balanced summary of what was done and what was found. | Four-paragraph structured abstract (Background, Methods, Results, Conclusion), below 300 words; reports the corrected candidate populations, budgets, semaglutide retail timing, and naltrexone/bupropion sensitivities. |
| INTRODUCTION | INTRODUCTION | INTRODUCTION |
| 2 | Background/rationale: explain the scientific background and rationale for the investigation being reported. | Introduction, paragraphs 1–2: global burden of obesity (>1 billion in 2022; doubling of adult prevalence and quadrupling of paediatric prevalence since 1990; GBD projection of 60% of adults with overweight or obesity by 2050; 4 million deaths attributable to elevated BMI in GBD 2015); Latin American context of nutritional transition and limited financing capacity. Introduction, paragraphs 3–4: Peruvian context (obesity prevalences of 23.23% by BMI, 38.90% by waist circumference, and 81.53% by waist-to-height ratio; 27.7% meeting criteria for pharmacotherapy; 2.5% for bariatric surgery); recognition of clinical heterogeneity; incorporation of modern pharmacotherapy (semaglutide, tirzepatide, SELECT trial) and the specific gap regarding population-level budget impact in Peru. |
| 3 | Objectives: state specific objectives, including any prespecified hypotheses. | Introduction, final paragraph: the study estimated survey-observable candidate populations and annual medication-acquisition costs for liraglutide, extended-release naltrexone/bupropion, exploratory extended-release phentermine/topiramate, and supplementary retail semaglutide 2.4 mg, with separate first-year and maintenance-year costs. Metformin and SGLT2 inhibitors were secondary cardiometabolic comparators. Structural sensitivity analyses evaluated candidate definitions, programmatic coverage, annual persistence, and price assumptions. Additional pharmacologic cost-source sensitivity analyses evaluated arithmetic naltrexone/bupropion costs and alternative semaglutide maintenance pricing. |
| METHODS | METHODS | METHODS |
| 4 | Study design: present key elements of study design early in the paper. | Methods, subsection «Study design»: cross-sectional observational analysis of secondary microdata from ENDES 2022–2024 combined with a deterministic budget impact model of pharmacologic acquisition costs. Explicit adherence to the STROBE recommendations for observational studies. |
| 5 | Setting: describe the setting, locations, and relevant dates, including periods of recruitment, exposure, follow-up, and data collection. | Methods, subsection «Data source»: ENDES is a continuous population-based survey conducted by INEI with a probabilistic, two-stage, stratified design independent by domains; one person aged 15 years or older per household is selected for interview and physical measurements; inferences are valid at the national, urban–rural, natural-region, and departmental levels. Period: annual rounds 2022–2024. Setting: nationwide (Peru). Original data collection conducted through in-person interviews with standardized procedures. |
| 6 | (a) Participants: give the eligibility criteria and the sources and methods of selection of participants. | Methods, subsection "Study population": adults aged 18 years or older with valid information for the variables required by each pharmacotherapy for obesity scenario; subpopulation of adults aged 30 years or older with diagnosed diabetes for secondary cardiometabolic comparator scenarios (SGLT2 inhibitors and metformin). Inclusion: valid information for the criterion of each scenario and for the corresponding auxiliary variables. Exclusion: non-response codes or invalid measurements treated as missing; data cleaning under physiologically plausible ranges (SBP 70–270 mmHg; DBP 30–150 mmHg; weight 25–300 kg; height 120–220 cm; waist 40–220 cm; BMI 10–80 kg/m²). No global participant deletion was performed for missing data; denominators vary across scenarios. Eligibility, BMI-observed, pooled-weight, diabetes-status, and weighted-contributor denominators are explicitly distinguished; Table S3 and Figure S1 show the complete flow. |
| 7 | Variables: clearly define all outcomes, exposures, predictors, potential confounders, and effect modifiers. Give diagnostic criteria, if applicable. | Methods, subsection "Variables and operational definitions". Clinical variables: BMI = weight (kg) / height (m)²; obesity ≥30; severe obesity ≥35; very severe obesity ≥40 kg/m². Measured hypertension: SBP ≥140 or DBP ≥90 mmHg; probable/treated hypertension: measured hypertension or self-reported use of antihypertensive medication. Diagnosed diabetes: self-reported diagnosis by a physician or other health worker. Primary pharmacotherapy for obesity scenarios (hierarchical): BMI-defined obesity; broad criterion (BMI ≥30 or BMI 27-<30 with comorbidity); pragmatic priority (BMI ≥35 or BMI 30-<35 with comorbidity); severe obesity; very severe obesity; diagnosed diabetes with BMI ≥27; diabetes with obesity. Secondary cardiometabolic comparator scenarios: SGLT2 inhibitors in diagnosed diabetes, including a risk-proxy scenario; metformin in diagnosed diabetes. SGLT2 inhibitors in isolated obesity served only as a nonclinical fiscal stress test. Stratum: self-reported health insurance (uninsured, SIS, EsSalud, others) as a stratum of potential budget pressure rather than a definitive administrative payer attribution. |
| 8* | Data sources/measurement: for each variable of interest, give sources of data and details of methods of assessment. Describe comparability of assessment methods if there is more than one group. | Methods, Data source and Medication-cost inputs: ENDES questionnaire, measured anthropometry and blood pressure, self-reported diabetes, treatment, sociodemographic characteristics, and insurance. Semaglutide presentation and dosing were verified using the DIGEMID technical sheet; complete retail titration prices and an alternative maintenance listing were documented with direct Zotero markers. Extended-release naltrexone/bupropion used the IETSI-reported annual cost as the main HTA scenario, while arithmetic maintenance-year and first-year dose-escalation costs were explicitly separated and supported by the CONTRAVE dosing source. Liraglutide and the remaining comparators retain their documented regulatory, HTA, or retail source hierarchy. |
| 9 | Bias: describe any efforts to address potential sources of bias. | Methods, subsection "Statistical analysis": incorporation of the complex sampling design (weights rescaled by division by 3 for the pooled file, unique cluster and stratum identifiers, variance estimation by Taylor linearization, single-PSU stratum handling through centering); design specification on the complete file and on subpopulations via svy: subpop to preserve the correct variance structure. Discussion, subsection "Limitations": explicit declaration of cross-sectional nature without temporality or causality; potential candidates observable in the survey rather than individual clinical eligibility; self-reported diabetes without discrimination between type 1 and type 2; insurance variable does not identify the actual payer; prices subject to procurement and market changes; liraglutide retail price treated as a high-cost non-governmental ceiling; and phentermine/topiramate analyzed only as exploratory because the Peruvian HTA did not approve EsSalud use. |
| 10 | Study size: explain how the study size was arrived at. | Methods, Study population, and Results 3.1. Study size was determined by frozen-data availability: 76,819 pooled records; 69,414 adults aged ≥18 years; 69,019 with pooled survey weight and complete design variables; 68,358 with BMI observed; and 68,043 with BMI plus pooled survey weight. The diabetes branch included 49,363 adults aged ≥30 years, 49,053 with pooled survey weight, and 49,011 with weight plus diabetes status. There were 2,599 diagnosed-diabetes observations, of whom 2,577 had pooled survey weight and contributed to weighted estimates. Figure S1 and Table S3 report the parallel denominators. |
| 11 | Quantitative variables: explain how quantitative variables were handled in the analyses. If applicable, describe which groupings were chosen and why. | Methods, subsection "Variables and operational definitions": BMI categorized by clinical thresholds (overweight 25-<30; obesity ≥30; severe ≥35; very severe ≥40 kg/m²); blood pressure dichotomized at the 140/90 mmHg cutoff; age used as an eligibility criterion per scenario (≥18 years for pharmacotherapy for obesity; ≥30 years for diabetes-restricted cardiometabolic comparator scenarios). Methods, subsection "Statistical analysis" (second paragraph): continuous variables summarized as weighted means with 95% CI; categorical variables as weighted proportions with 95% CI. |
| 12 | (a) Statistical methods: describe all statistical methods, including those used to control for confounding. | Methods, subsection «Statistical analysis»: estimation of unweighted counts, weighted proportions, weighted population totals, and 95% CI for each scenario, with denominators specific to anthropometric requirements or self-reported information. Budget impact model: projected annual cost = N candidates × annual cost per patient × programme coverage × annual persistence. Programme coverage was assessed at 20%, 50%, 80%, and 100%; annual persistence was set at 100% in the principal scenario as a direct budget ceiling. Explicit rationale (fourth paragraph): adjusted regression models were not used as the principal analysis because the objective was to quantify absolute populations and costs rather than to estimate aetiological associations. Multivariable adjustment would have shifted the estimand toward a causal question that was not posed. |
|  | (b) Describe any methods used to examine subgroups and interactions. | Methods, subsection "Statistical analysis", and Results, subsection "Distribution by self-reported health insurance" (Figure 2 and Table S4): stratification by self-reported insurance (uninsured, SIS, EsSalud, others) as subgroups of potential budget pressure. Results, subsection "Structural sensitivity and internal stability" (Figure 3): structural sensitivity of the high-cost non-governmental liraglutide 3.0 mg pragmatic-priority scenario across variation in population size, coverage, annual unit cost, annual persistence, and hypertension/comorbidity definition. |
|  | (c) Explain how missing data were addressed. | Methods, subsection «Study population»: no global deletion for missing data; each participant contributed to the analyses for which valid information was available; non-response codes or invalid measurements treated as missing. Results, subsection «Participant selection and general characteristics», and Table S3: scenario-specific denominators; BMI-based criteria require valid anthropometry, whereas scenarios based on diagnosed diabetes do not lose observations from absent anthropometry when it is not part of the criterion. |
|  | (d) If applicable, explain how the study design takes account of the sampling strategy. | Methods, subsection «Statistical analysis» (first paragraph): incorporation of weights, strata, and primary sampling units of ENDES. Weight rescaling by division by 3 for the pooled file; QHCLUSTER as PSU, HV022 as stratum; variance estimation by Taylor linearization; centring for strata with a single PSU; use of svy: subpop on the complete file to preserve the variance structure when estimating across subpopulations. |
|  | (e) Describe any sensitivity analyses. | Methods, subsection "Statistical analysis" (penultimate paragraph): substitution of probable/treated hypertension by measured hypertension only; restriction of overweight-comorbidity to diagnosed diabetes; adiposity estimates by elevated waist circumference and waist-to-height ratio ≥0.50 (not as principal pharmacotherapy criteria); annual stability analyses for 2022, 2023, and 2024. Results, subsection "Structural sensitivity and internal stability" (Figure 3, Tables S5-S6, Figure S3): range of variation across population definition, coverage, price, persistence, and stricter hypertension definition; annual stability of 27.6%-28.5% (BMI-defined obesity), 33.2%-34.2% (broad eligibility criterion for obesity medication), 12.8%-14.4% (pragmatic priority), 4.9%-5.2% (SGLT2 risk-proxy comparator), and 7.6%-7.7% (metformin comparator). |
| RESULTS | RESULTS | RESULTS |
| 13* | (a) Participants: report the numbers of individuals at each stage of the study (e.g., numbers potentially eligible, examined for eligibility, confirmed eligible, included in the study, completing follow-up, and analysed). | Results 3.1, Figure S1, and Table S3. Pooled file: 76,819 records; adults aged ≥18 years: 69,414; pooled-weight adult contributors: 69,019; BMI observed: 68,358; BMI plus pooled weight: 68,043. Diabetes branch: 49,363 adults aged ≥30 years; 49,053 with pooled weight; 49,011 with pooled weight and diabetes status; 2,599 diagnosed-diabetes observations; 2,577 weighted diagnosed-diabetes contributors; 22 diagnoses with weight missing; and 42 records with weight observed but diabetes status missing. |
|  | (b) Give reasons for non-participation at each stage. | Not applicable as a stage-by-stage flow: ENDES is a continuous population survey with response rates reported by INEI at the national level. The denominators reported per scenario reflect missingness on the relevant variables rather than refusals at recruitment. |
|  | (c) Consider use of a flow diagram. | Figure S1: parallel-denominator flow diagram showing the analytic sample by scenario branch (BMI-based pharmacotherapy for obesity vs. diabetes-restricted secondary cardiometabolic comparator scenarios). |
| 14* | (a) Descriptive data: give characteristics of study participants (demographic, clinical, social) and information on exposures and potential confounders. | Results, subsection «Participant selection and general characteristics», and Table 1. Weighted mean age 43.2 years (95% CI: 43.0–43.4); 51.0% women; 83.5% urban. Insurance coverage: 55.3% SIS, 25.4% EsSalud, 14.7% uninsured. Mean BMI 27.7 kg/m² (95% CI: 27.6–27.8); 69.3% overweight or obese; 28.2% obese by BMI ≥30; 73.9% elevated waist circumference; 87.3% waist-to-height ratio ≥0.50. Diagnosed diabetes 5.8%; probable/treated hypertension 19.5%. |
|  | (b) Indicate the number of participants with missing data for each variable of interest. | Table S3: missingness by variable across the three ENDES rounds, with per-variable denominators displayed alongside each scenario estimate in Table 2. |
| 15* | Outcome data: report numbers of outcome events or summary measures. | Results, Potential candidates for treatment with obesity medications, and Table 2. A total of 20,300 participants met the broad criterion among 68,030 adults with the required data. The weighted estimate was 33.8%, corresponding to 9.04 million adults. BMI-defined obesity represented 7.54 million; pragmatic priority, 3.67 million; severe obesity, 1.99 million; and BMI ≥40 kg/m², 0.52 million. Among diabetes-related scenarios, diabetes with obesity represented 0.60 million adults. Semaglutide and the secondary cardiometabolic comparator results are reported separately. |
| 16 | (a) Main results: give unadjusted estimates and, if applicable, confounder-adjusted estimates and their precision (e.g., 95% CI). Make clear which confounders were adjusted for and why they were included. | Results, subsection "Projected budget impact", and Figure 1. At 100% coverage under the high-cost non-governmental liraglutide 3.0 mg scenario: broad eligibility criterion for obesity medication S/ 142.95 billion; pragmatic priority S/ 57.95 billion; severe obesity S/ 31.47 billion; BMI ≥40 S/ 8.25 billion; diabetes with obesity S/ 9.44 billion. Extended-release naltrexone/bupropion yielded S/ 23.65 billion under the broad criterion and S/ 9.59 billion under pragmatic priority. Exploratory extended-release phentermine/topiramate yielded S/ 26.83 billion and S/ 10.88 billion, respectively. Secondary cardiometabolic comparators at 100% coverage: S/ 2.00 billion for dapagliflozin in diabetes with risk proxy, S/ 2.48 billion for empagliflozin 10 mg, S/ 3.48 billion for empagliflozin 25 mg, and S/ 0.066 billion for metformin in diagnosed diabetes. Justification for the absence of multivariable adjustment in Methods, subsection "Statistical analysis" (fourth paragraph): the objective is to quantify populations and costs, not to estimate independent effects. Semaglutide costs are S/ 16,804.86 in the first year and S/ 18,135.52 in maintenance; Table S10 reports the IETSI main naltrexone/bupropion scenario and both arithmetic sensitivities. |
|  | (b) Report category boundaries when continuous variables were categorized. | BMI cutpoints (≥25, ≥27, ≥30, ≥35, ≥40 kg/m²), age cutpoints (≥18 and ≥30 years), and blood pressure cutpoints (140/90 mmHg) reported in Methods «Variables and operational definitions» and reproduced in Table 1 and Table 2. |
|  | (c) If applicable, consider translating estimates of relative risk into absolute risk for a meaningful time period. | Results, subsection «Projected budget impact», and Figure 1: estimates are reported directly on the absolute scale (population totals and annual costs in Peruvian soles), given the nature of the estimand. Methods, subsection «Statistical analysis»: estimation of weighted proportions and population totals with 95% CI for each scenario; relative measures that do not apply to a budget impact estimand were avoided. |
| 17 | Other analyses: report other analyses done (e.g., analyses of subgroups, interactions, and sensitivity analyses). | Results 3.4, Figure 3, and Tables S4–S6 and S10. Insurance-stratified and structural sensitivity analyses examined candidate definitions, coverage, annual cost, persistence, and comorbidity definitions. Table S10 reports semaglutide first-year, primary maintenance-year, and alternative maintenance-price scenarios, together with the IETSI-reported naltrexone/bupropion annual-cost scenario and the arithmetic maintenance-year and first-year dose-escalation sensitivities. |
| DISCUSSION | DISCUSSION | DISCUSSION |
| 18 | Key results: summarise key results with reference to study objectives. | Discussion, subsection "Principal findings" (first paragraph). The Peruvian adult population meeting survey-observable criteria for pharmacotherapy for obesity is large and sensitive to the prioritization rule: 9.04 million under the broad criterion, 7.54 million by BMI-defined obesity, and 3.67 million under the pragmatic-priority rule. When these populations were costed using liraglutide 3.0 mg as a high-cost non-governmental ceiling scenario, projected annual costs remained large. Extended-release naltrexone/bupropion produced lower but still nontrivial budget impact estimates. Extended-release phentermine/topiramate was analyzed only as an exploratory comparator. Secondary cardiometabolic comparator analyses with SGLT2 inhibitors and metformin illustrated the affordability gradient but were not interpreted as primary pharmacotherapy for obesity scenarios. |
| 19 | Limitations: discuss limitations of the study, taking into account sources of potential bias or imprecision. Discuss both direction and magnitude of any potential bias. | Discussion, Limitations: exactly two replacement paragraphs distinguish survey-observable candidates from individual clinical eligibility; self-reported diabetes, treatment, and insurance; missing eligibility variables; insurance from actual payer; retail from institutional prices; semaglutide first-year from maintenance; the IETSI-versus-arithmetic naltrexone/bupropion discrepancy; verified tirzepatide and orlistat retail observations that were not costed; the reference scenario; excluded untreated-obesity costs; and excluded monitoring, adverse events, discontinuation, avoided complications, savings, outcomes, net budget impact, and cost-effectiveness. |
| 20 | Interpretation: give a cautious overall interpretation of results considering objectives, limitations, multiplicity of analyses, results from similar studies, and other relevant evidence. | Discussion, subsections "Comparison with other studies" and "Implications for public and international health". Comparison: 28.2% obesity by BMI consistent with the previous Peruvian review reporting 23.23%; 33.8% from the broad pharmacotherapy for obesity scenario higher than the 27.7% reported in 2014–2022 surveys. Discussion of clinical obesity vs. BMI-defined obesity; STEP 1, SURMOUNT-1, and SELECT trials as clinical rationale for modern pharmacotherapy for obesity (STEP 1, SURMOUNT-1, and SELECT); Canadian and US models of fiscal impact for GLP-1 receptor agonists in obesity; gap between market prices and production costs. Cautious interpretation: scenarios represent observable candidates and budget ceilings, not individual clinical eligibility nor official quotations (Discussion, subsection "Limitations"). |
| 21 | Generalisability: discuss the generalisability (external validity) of the study results. | Discussion, subsection "Implications for public and international health" (paragraphs 2–3): applicability to low- and middle-income countries with simultaneous rises in obesity, cardiometabolic transition, constrained budgets, and social pressure to incorporate modern obesity-treatment therapies. Contribution of a reproducible framework: define observable candidates, separate pharmacotherapy for obesity scenarios from secondary cardiometabolic comparators and fiscal stress tests, and cross-tabulate population, price, coverage, and persistence before committing to coverage decisions. Population-level inference is restricted to the 2022–2024 period and to the prevailing price and coverage structure. |
| OTHER INFORMATION | OTHER INFORMATION | OTHER INFORMATION |
| 22 | Funding: give the source of funding and the role of the funders for the present study and, if applicable, for the original study on which the present article is based. | Declarations, Funding: This study was self-funded and received no specific grant from any funding agency in the public, commercial, or not-for-profit sectors. No external funder had any role in study design, analysis, interpretation, or reporting. |

*STROBE = Strengthening the Reporting of Observational Studies in Epidemiology. Items specific to cohort or case-control studies do not apply to this cross-sectional analysis.*

Table S2. Variable definitions and analytic use.

| **Variable** | **Definition** | **Analytic use** |
| --- | --- | --- |
| Adult population | Age ≥18 years in ENDES 2022–2024 | Base for pharmacotherapy for obesity scenarios |
| Diabetes population | Age ≥30 years and self-reported diagnosed diabetes | Base for secondary cardiometabolic comparators and diabetes-related obesity-treatment subgroups |
| Obesity | BMI ≥30 kg/m² | BMI-defined pharmacotherapy for obesity criterion |
| Broad pharmacotherapy for obesity criterion | BMI ≥30 kg/m² or BMI 27-<30 kg/m² with diabetes or probable/treated hypertension | Broad pharmacotherapy for obesity scenario |
| Pragmatic-priority pharmacotherapy for obesity criterion | BMI ≥35 kg/m² or BMI 30-<35 kg/m² with diabetes or probable/treated hypertension | Stepped-implementation pharmacotherapy for obesity scenario |
| Secondary SGLT2-inhibitor cardiometabolic comparator | Diagnosed diabetes at age ≥30 years with obesity or probable/treated hypertension | ENDES proxy for high cardiometabolic risk; not an indication for obesity treatment |
| Secondary metformin cardiometabolic comparator | Diagnosed diabetes at age ≥30 years | Low-cost diabetes comparator; not an obesity-medication scenario |
| Probable/treated hypertension | BP ≥140/90 mmHg or use of antihypertensive medication | Observable comorbidity for risk scenarios |
| Reported insurance | Uninsured, SIS, EsSalud, other | Analytic stratum of budgetary pressure |

*BMI = body mass index; BP = blood pressure; SGLT2 = sodium–glucose cotransporter 2; SIS = Seguro Integral de Salud.*

Table S3. Missingness and analytic denominators.

| **Variable** | **Non-missing or count** | **Missing or interpretation** |
| --- | --- | --- |
| Age | 76,818 | 1 |
| Sex | 76,819 | 0 |
| Health insurance | 76,816 | 3 |
| BMI | 75,637 | 1182 |
| Waist circumference | 74,778 | 2041 |
| Waist-to-height ratio | 74,757 | 2062 |
| SBP | 76,291 | 528 |
| DBP | 76,304 | 515 |
| Diagnosed diabetes | 76,747 | 72 |
| Probable/treated hypertension | 76,320 | 499 |
| Observable comorbidity | 76,283 | 536 |
| Purchase of diabetes medications | 2868 | 73,951 |
| Purchase with prescription | 2030 | 74,789 |
| Self-reported adherence | 2038 | 74,781 |
| pharmacotherapy for obesity candidate, BMI ≥30 kg/m² | 68,043 | 8776 |
| Broad pharmacotherapy for obesity candidate | 68,030 | 8789 |
| Pragmatic-priority pharmacotherapy for obesity candidate | 68,034 | 8785 |
| Secondary metformin comparator candidate | 49,011 | 27,808 |
| Secondary SGLT2-inhibitor proxy comparator candidate | 48,975 | 27,844 |
| Adults aged ≥18 years | 69,414 | Source branch |
| BMI observed among adults aged ≥18 years | 68,358 | Observed clinical information |
| BMI + pooled survey weight | 68,043 | Design-weighted BMI denominator |
| Adults aged ≥30 years | 49,363 | Diabetes source population |
| Adults aged ≥30 years with non-missing pooled survey weight | 49,053 | Survey-weight availability |
| Adults aged ≥30 years with non-missing pooled survey weight and non-missing diabetes status | 49,011 | Design-weighted diabetes denominator |
| Diagnosed diabetes irrespective of survey-weight availability | 2,599 | Observed diagnoses |
| Diagnosed diabetes with non-missing pooled survey weight | 2,577 | Contributed to weighted estimates |
| Diagnosed diabetes with pooled survey weight missing | 22 | Excluded from weighted estimates |
| Pooled survey weight observed but diabetes status missing | 42 | Excluded from diabetes denominator |

*Source records, observed clinical information, and survey-weighted denominators are presented separately. BMI = body mass index; DBP = diastolic blood pressure; SBP = systolic blood pressure; SGLT2 = sodium–glucose cotransporter 2.*

Table S4. Candidate populations by insurance category.

| **Scenario** | **Insurance** | **n/N** | **% within insurance (95% CI)** | **Estimated population, millions (95% CI)** |
| --- | --- | --- | --- | --- |
| pharmacotherapy for obesity: BMI ≥30 kg/m² | Uninsured | 2181/8225 | 28.4% (26.7–30.0%) | 1.11 (1.03–1.19) |
| pharmacotherapy for obesity: BMI ≥30 kg/m² | SIS | 10,858/45,105 | 26.6% (25.8–27.3%) | 3.94 (3.79–4.09) |
| pharmacotherapy for obesity: BMI ≥30 kg/m² | EsSalud | 3950/13,027 | 31.0% (29.6–32.4%) | 2.10 (1.97–2.22) |
| pharmacotherapy for obesity: BMI ≥30 kg/m² | Other | 537/1686 | 31.2% (27.8–34.6%) | 0.39 (0.33–0.44) |
| pharmacotherapy for obesity: broad criterion | Uninsured | 2534/8224 | 33.6% (31.8–35.3%) | 1.31 (1.23–1.40) |
| pharmacotherapy for obesity: broad criterion | SIS | 12,371/45,097 | 31.0% (30.2–31.8%) | 4.60 (4.43–4.76) |
| pharmacotherapy for obesity: broad criterion | EsSalud | 4762/13,023 | 39.2% (37.8–40.7%) | 2.65 (2.50–2.80) |
| pharmacotherapy for obesity: broad criterion | Other | 633/1686 | 38.6% (34.7–42.5%) | 0.48 (0.42–0.54) |
| pharmacotherapy for obesity: pragmatic priority | Uninsured | 980/8224 | 13.8% (12.5–15.0%) | 0.54 (0.48–0.59) |
| pharmacotherapy for obesity: pragmatic priority | SIS | 4397/45,099 | 12.5% (11.9–13.1%) | 1.85 (1.75–1.95) |
| pharmacotherapy for obesity: pragmatic priority | EsSalud | 1900/13,025 | 16.4% (15.3–17.4%) | 1.11 (1.02–1.19) |
| pharmacotherapy for obesity: pragmatic priority | Other | 232/1686 | 13.7% (11.3–16.1%) | 0.17 (0.14–0.20) |
| pharmacotherapy for obesity: severe obesity | Uninsured | 565/8225 | 7.5% (6.6–8.4%) | 0.29 (0.26–0.33) |
| pharmacotherapy for obesity: severe obesity | SIS | 2781/45,105 | 7.3% (6.8–7.7%) | 1.08 (1.01–1.15) |
| pharmacotherapy for obesity: severe obesity | EsSalud | 999/13,027 | 7.8% (7.1–8.6%) | 0.53 (0.48–0.58) |
| pharmacotherapy for obesity: severe obesity | Other | 112/1686 | 7.0% (5.2–8.7%) | 0.09 (0.06–0.11) |
| pharmacotherapy for obesity: very severe obesity | Uninsured | 150/8225 | 2.2% (1.6–2.8%) | 0.09 (0.06–0.11) |
| pharmacotherapy for obesity: very severe obesity | SIS | 642/45,105 | 1.8% (1.6–2.1%) | 0.27 (0.24–0.31) |
| pharmacotherapy for obesity: very severe obesity | EsSalud | 246/13,027 | 2.1% (1.7–2.5%) | 0.14 (0.12–0.17) |
| pharmacotherapy for obesity: very severe obesity | Other | 22/1686 | 1.6% (0.8–2.5%) | 0.02 (0.01–0.03) |
| pharmacotherapy for obesity: diagnosed diabetes + BMI ≥27 | Uninsured | 146/5725 | 3.5% (2.6–4.4%) | 0.09 (0.07–0.12) |
| pharmacotherapy for obesity: diagnosed diabetes + BMI ≥27 | SIS | 809/30,296 | 4.2% (3.8–4.7%) | 0.43 (0.38–0.48) |
| pharmacotherapy for obesity: diagnosed diabetes + BMI ≥27 | EsSalud | 571/10,888 | 6.5% (5.7–7.3%) | 0.37 (0.32–0.42) |
| pharmacotherapy for obesity: diagnosed diabetes + BMI ≥27 | Other | 72/1401 | 7.0% (4.7–9.2%) | 0.07 (0.05–0.10) |
| pharmacotherapy for obesity: diagnosed diabetes + obesity | Uninsured | 89/5725 | 2.3% (1.5–3.1%) | 0.06 (0.04–0.08) |
| pharmacotherapy for obesity: diagnosed diabetes + obesity | SIS | 499/30,296 | 2.7% (2.4–3.1%) | 0.28 (0.24–0.32) |
| pharmacotherapy for obesity: diagnosed diabetes + obesity | EsSalud | 352/10,888 | 3.9% (3.2–4.6%) | 0.22 (0.18–0.26) |
| pharmacotherapy for obesity: diagnosed diabetes + obesity | Other | 41/1401 | 3.5% (1.8–5.1%) | 0.04 (0.02–0.05) |
| Secondary cardiometabolic comparator: metformin, diagnosed diabetes | Uninsured | 231/5816 | 5.5% (4.4–6.7%) | 0.15 (0.12–0.18) |
| Secondary cardiometabolic comparator: metformin, diagnosed diabetes | SIS | 1346/30,663 | 6.5% (6.0–7.0%) | 0.67 (0.61–0.72) |
| Secondary cardiometabolic comparator: metformin, diagnosed diabetes | EsSalud | 904/11,095 | 10.3% (9.3–11.3%) | 0.60 (0.53–0.66) |
| Secondary cardiometabolic comparator: metformin, diagnosed diabetes | Other | 96/1437 | 9.1% (6.7–11.6%) | 0.10 (0.07–0.13) |
| Secondary cardiometabolic comparator: metformin, diagnosed diabetes + obesity | Uninsured | 89/5725 | 2.3% (1.5–3.1%) | 0.06 (0.04–0.08) |
| Secondary cardiometabolic comparator: metformin, diagnosed diabetes + obesity | SIS | 499/30,296 | 2.7% (2.4–3.1%) | 0.28 (0.24–0.32) |
| Secondary cardiometabolic comparator: metformin, diagnosed diabetes + obesity | EsSalud | 352/10,888 | 3.9% (3.2–4.6%) | 0.22 (0.18–0.26) |
| Secondary cardiometabolic comparator: metformin, diagnosed diabetes + obesity | Other | 41/1401 | 3.5% (1.8–5.1%) | 0.04 (0.02–0.05) |
| Secondary cardiometabolic comparator: SGLT2 inhibitor, diagnosed diabetes | Uninsured | 231/5816 | 5.5% (4.4–6.7%) | 0.15 (0.12–0.18) |
| Secondary cardiometabolic comparator: SGLT2 inhibitor, diagnosed diabetes | SIS | 1346/30,663 | 6.5% (6.0–7.0%) | 0.67 (0.61–0.72) |
| Secondary cardiometabolic comparator: SGLT2 inhibitor, diagnosed diabetes | EsSalud | 904/11,095 | 10.3% (9.3–11.3%) | 0.60 (0.53–0.66) |
| Secondary cardiometabolic comparator: SGLT2 inhibitor, diagnosed diabetes | Other | 96/1437 | 9.1% (6.7–11.6%) | 0.10 (0.07–0.13) |
| Secondary cardiometabolic comparator: SGLT2 inhibitor, diabetes + risk proxy | Uninsured | 136/5813 | 3.5% (2.6–4.5%) | 0.10 (0.07–0.12) |
| Secondary cardiometabolic comparator: SGLT2 inhibitor, diabetes + risk proxy | SIS | 815/30,645 | 4.2% (3.8–4.7%) | 0.43 (0.38–0.48) |
| Secondary cardiometabolic comparator: SGLT2 inhibitor, diabetes + risk proxy | EsSalud | 598/11,082 | 7.1% (6.2–8.0%) | 0.41 (0.36–0.47) |
| Secondary cardiometabolic comparator: SGLT2 inhibitor, diabetes + risk proxy | Other | 69/1435 | 6.7% (4.4–8.9%) | 0.07 (0.05–0.10) |
| Secondary SGLT2-inhibitor fiscal stress test: isolated obesity (nonclinical) | Uninsured | 2181/8225 | 28.4% (26.7–30.0%) | 1.11 (1.03–1.19) |
| Secondary SGLT2-inhibitor fiscal stress test: isolated obesity (nonclinical) | SIS | 10,858/45,105 | 26.6% (25.8–27.3%) | 3.94 (3.79–4.09) |
| Secondary SGLT2-inhibitor fiscal stress test: isolated obesity (nonclinical) | EsSalud | 3950/13,027 | 31.0% (29.6–32.4%) | 2.10 (1.97–2.22) |
| Secondary SGLT2-inhibitor fiscal stress test: isolated obesity (nonclinical) | Other | 537/1686 | 31.2% (27.8–34.6%) | 0.39 (0.33–0.44) |

*BMI = body mass index; CI = confidence interval; SIS = Seguro Integral de Salud; SGLT2 = sodium–glucose cotransporter 2. Insurance categories indicate potential pressure strata, not definitive payer responsibility.*

Table S5. Annual stability of scenario estimates.

| **Scenario** | **Year** | **n/N** | **Weighted % (95% CI)** |
| --- | --- | --- | --- |
| pharmacotherapy for obesity: BMI ≥30 kg/m² | 2022 | 5193/19,775 | 28.5% (27.4–29.5%) |
| pharmacotherapy for obesity: BMI ≥30 kg/m² | 2023 | 5896/23,274 | 27.6% (26.6–28.6%) |
| pharmacotherapy for obesity: BMI ≥30 kg/m² | 2024 | 6437/24,994 | 28.5% (27.4–29.5%) |
| pharmacotherapy for obesity: broad criterion | 2022 | 6019/19,772 | 34.0% (32.9–35.1%) |
| pharmacotherapy for obesity: broad criterion | 2023 | 6801/23,269 | 33.2% (32.1–34.3%) |
| pharmacotherapy for obesity: broad criterion | 2024 | 7480/24,989 | 34.2% (33.2–35.3%) |
| pharmacotherapy for obesity: pragmatic priority | 2022 | 2251/19,774 | 14.4% (13.5–15.2%) |
| pharmacotherapy for obesity: pragmatic priority | 2023 | 2444/23,269 | 12.8% (12.0–13.5%) |
| pharmacotherapy for obesity: pragmatic priority | 2024 | 2814/24,991 | 14.0% (13.2–14.9%) |
| Secondary cardiometabolic comparator: SGLT2 inhibitor, diabetes + risk proxy | 2022 | 480/13,895 | 5.2% (4.6–5.9%) |
| Secondary cardiometabolic comparator: SGLT2 inhibitor, diabetes + risk proxy | 2023 | 565/16,952 | 5.2% (4.5–5.9%) |
| Secondary cardiometabolic comparator: SGLT2 inhibitor, diabetes + risk proxy | 2024 | 573/18,128 | 4.9% (4.3–5.6%) |
| Secondary cardiometabolic comparator: metformin, diagnosed diabetes | 2022 | 775/13,908 | 7.6% (6.8–8.4%) |
| Secondary cardiometabolic comparator: metformin, diagnosed diabetes | 2023 | 876/16,958 | 7.7% (6.9–8.5%) |
| Secondary cardiometabolic comparator: metformin, diagnosed diabetes | 2024 | 926/18,145 | 7.6% (6.8–8.3%) |

*BMI = body mass index; CI = confidence interval; ENDES = Peruvian Demographic and Family Health Survey; SGLT2 = sodium–glucose cotransporter 2.*

Table S6. Structural sensitivity definitions.

| **Sensitivity definition** | **n/N** | **Weighted % (95% CI)** | **Estimated population, millions (95% CI)** | **Interpretation** |
| --- | --- | --- | --- | --- |
| Main broad pharmacotherapy for obesity definition | 20,300/68,030 | 33.8% (33.1–34.5%) | 9.04 (8.75–9.33) | Candidate definition |
| Broad pharmacotherapy for obesity, measured hypertension only | 19,942/68,043 | 33.1% (32.4–33.8%) | 8.85 (8.56–9.13) | Candidate definition |
| Broad pharmacotherapy for obesity, diabetes comorbidity only | 18,178/68,043 | 29.6% (28.9–30.2%) | 7.91 (7.65–8.18) | Candidate definition |
| Main pragmatic-priority pharmacotherapy for obesity definition | 7509/68,034 | 13.7% (13.2–14.2%) | 3.67 (3.50–3.83) | Candidate definition |
| Pragmatic-priority pharmacotherapy for obesity, measured hypertension only | 7165/68,043 | 13.1% (12.6–13.5%) | 3.49 (3.34–3.65) | Candidate definition |
| Adiposity by elevated waist circumference, IDF criterion | 48,205/67,227 | 73.9% (73.4–74.5%) | 19.61 (19.12–20.11) | Adiposity burden only; not a main pharmacologic criterion |
| Adiposity by waist-to-height ratio | 58,657/67,208 | 87.3% (86.9–87.8%) | 23.17 (22.61–23.72) | Adiposity burden only; not a main pharmacologic criterion |

*BMI = body mass index; CI = confidence interval; IDF = International Diabetes Federation.*

Table S7. Medication price-source and costing-decision matrix.

| **Class** | **Medication** | **Presentation** | **Unit price** | **Annual cost or modeled annual assumption** | **Status** | **Decision** |
| --- | --- | --- | --- | --- | --- | --- |
| Biguanide | Metformin hydrochloride | 850 mg tablet | S/ 0.06 | S/ 43.80 | Complete | Secondary low-cost comparator |
| Biguanide | Metformin hydrochloride | 500 mg tablet | S/ 0.25 | S/ 182.50 | Complete | Dose sensitivity only |
| SGLT2 inhibitor | Dapagliflozin | 10 mg tablet | S/ 5.40 | S/ 1,971.00 | Complete | Secondary comparator base |
| SGLT2 inhibitor | Empagliflozin | 25 mg tablet | S/ 9.40 | S/ 3,431.00 | Complete | Secondary comparator alternative |
| SGLT2 inhibitor | Empagliflozin | 10 mg tablet | S/ 6.70 | S/ 2,445.50 | Complete | Secondary comparator alternative |
| GLP-1 | Liraglutide | Saxenda 6 mg/mL, 18 mg/3 mL pen | S/ 259.90/pen | S/ 15,810.57 | Complete retail | Primary non-governmental ceiling scenario |
| Non-GLP-1 obesity treatment | Naltrexone/bupropion | 8 mg/90 mg ER | S/ 1.35/tablet | S/ 2,615.90 | Complete HTA (27) | Main non-GLP-1 comparator; arithmetic sensitivities in S10 |
| Non-GLP-1 obesity treatment | Phentermine/topiramate | ER formulation | S/ 8.13 | S/ 2,967.45 | Complete HTA; exploratory (28) | Supplementary comparator; not a coverage recommendation |
| GLP-1 | Semaglutide 2.4 mg | Five-strength four-dose pens | S/ 840.50–1,395.04/pen | S/ 16,804.86 first year; S/ 18,135.52 maintenance | Complete retail and regulatory documentation (34–40) | Supplementary non-governmental retail scenario |
| GIP/GLP-1 | Tirzepatide | Mounjaro KwikPen 2.5 mg and 7.5 mg listings | S/ 1,399 and S/ 2,099 | Not costed | Selected retail strengths verified (44,45) | Complete indication-concordant annual regimen not established |
| Lipase inhibitor | Orlistat | 120 mg capsules, box of 30 | S/ 90/box | Not costed | Retail observation verified (46) | Complete reproducible annual regimen not established |

*ER = extended release; GIP = glucose-dependent insulinotropic polypeptide; GLP-1 = glucagon-like peptide-1; HTA = health technology assessment; IETSI = Instituto de Evaluación de Tecnologías en Salud e Investigación; SGLT2 = sodium–glucose cotransporter 2. Price sources and decisions are supported by references 22, 23, 25–28, and 34–46. Tirzepatide and orlistat were not costed because a complete indication-concordant annual regimen was not established.*

Table S8. Annual national acquisition budgets by coverage level (S/ billions).

| **Scenario** | **20%** | **50%** | **80%** | **100%** |
| --- | --- | --- | --- | --- |
| Liraglutide 3.0 mg, broad eligibility criterion | 28.59 | 71.48 | 114.36 | 142.95 |
| Liraglutide 3.0 mg, pragmatic priority | 11.59 | 28.97 | 46.36 | 57.95 |
| Liraglutide 3.0 mg, severe obesity | 6.29 | 15.73 | 25.17 | 31.47 |
| Liraglutide 3.0 mg, very severe obesity | 1.65 | 4.12 | 6.60 | 8.25 |
| Liraglutide 3.0 mg, diabetes + obesity | 1.89 | 4.72 | 7.55 | 9.44 |
| Naltrexone/bupropion ER, broad eligibility criterion | 4.73 | 11.83 | 18.92 | 23.65 |
| Naltrexone/bupropion ER, pragmatic priority | 1.92 | 4.79 | 7.67 | 9.59 |
| Phentermine/topiramate ER, broad eligibility criterion | 5.37 | 13.42 | 21.46 | 26.83 |
| Phentermine/topiramate ER, pragmatic priority | 2.18 | 5.44 | 8.70 | 10.88 |
| SGLT2-inhibitor proxy, dapagliflozin | 0.40 | 1.00 | 1.60 | 2.00 |
| SGLT2-inhibitor proxy, empagliflozin 10 mg | 0.50 | 1.24 | 1.98 | 2.48 |
| SGLT2-inhibitor proxy, empagliflozin 25 mg | 0.70 | 1.74 | 2.78 | 3.48 |
| Metformin, diagnosed diabetes | 0.01 | 0.03 | 0.05 | 0.07 |
| Semaglutide 2.4 mg, broad, first year | 30.39 | 75.97 | 121.55 | 151.94 |
| Semaglutide 2.4 mg, broad, maintenance | 32.79 | 81.99 | 131.18 | 163.97 |
| Semaglutide 2.4 mg, pragmatic priority, first year | 12.32 | 30.79 | 49.27 | 61.59 |
| Semaglutide 2.4 mg, pragmatic priority, maintenance | 13.29 | 33.23 | 53.17 | 66.47 |

*Budgets use unrounded survey-weighted populations and annual per-patient costs; all national budgets are rounded to two decimals. ER = extended release; SGLT2 = sodium–glucose cotransporter 2.*

Table S9. Annual acquisition budget by insurance pressure stratum.

| **Scenario** | **Insurance** | **Candidates, millions** | **Annual cost, S/ billions** |
| --- | --- | --- | --- |
| Liraglutide 3.0 mg, pragmatic priority | Uninsured | 0.54 | 8.51 |
| Liraglutide 3.0 mg, pragmatic priority | SIS | 1.85 | 29.26 |
| Liraglutide 3.0 mg, pragmatic priority | EsSalud | 1.11 | 17.50 |
| Liraglutide 3.0 mg, pragmatic priority | Other | 0.17 | 2.68 |
| Naltrexone/bupropion ER, pragmatic priority | Uninsured | 0.54 | 1.41 |
| Naltrexone/bupropion ER, pragmatic priority | SIS | 1.85 | 4.84 |
| Naltrexone/bupropion ER, pragmatic priority | EsSalud | 1.11 | 2.89 |
| Naltrexone/bupropion ER, pragmatic priority | Other | 0.17 | 0.44 |
| Phentermine/topiramate ER, pragmatic priority | Uninsured | 0.54 | 1.60 |
| Phentermine/topiramate ER, pragmatic priority | SIS | 1.85 | 5.49 |
| Phentermine/topiramate ER, pragmatic priority | EsSalud | 1.11 | 3.28 |
| Phentermine/topiramate ER, pragmatic priority | Other | 0.17 | 0.50 |
| Secondary SGLT2-inhibitor proxy, dapagliflozin | Uninsured | 0.10 | 0.20 |
| Secondary SGLT2-inhibitor proxy, dapagliflozin | SIS | 0.43 | 0.85 |
| Secondary SGLT2-inhibitor proxy, dapagliflozin | EsSalud | 0.41 | 0.81 |
| Secondary SGLT2-inhibitor proxy, dapagliflozin | Other | 0.07 | 0.14 |
| Secondary metformin comparator, diagnosed diabetes | Uninsured | 0.15 | 0.01 |
| Secondary metformin comparator, diagnosed diabetes | SIS | 0.67 | 0.03 |
| Secondary metformin comparator, diagnosed diabetes | EsSalud | 0.60 | 0.03 |
| Secondary metformin comparator, diagnosed diabetes | Other | 0.10 | 0.00 |

*ER = extended release; SGLT2 = sodium–glucose cotransporter 2; SIS = Seguro Integral de Salud. Insurance strata identify where potential budget pressure may concentrate; they do not assign actual payer responsibility. National budgets are rounded to two decimals.*

Table S10. Pharmacologic cost-source sensitivity analyses.

| **Panel** | **Cost scenario** | **Annual cost/patient** | **Population scenario** | **Budget at 100%, S/ bn** | **Difference, S/ bn** | **Difference, %** |
| --- | --- | --- | --- | --- | --- | --- |
| A. Semaglutide 2.4 mg | First-year full titration | S/ 16,804.86 | Broad criterion | 151.94 | -12.03 | -7.34% |
| A. Semaglutide 2.4 mg | First-year full titration | S/ 16,804.86 | Pragmatic priority | 61.59 | -4.88 | -7.34% |
| A. Semaglutide 2.4 mg | Maintenance year, primary | S/ 18,135.52 | Broad criterion | 163.97 | +0.00 | +0.00% |
| A. Semaglutide 2.4 mg | Maintenance year, primary | S/ 18,135.52 | Pragmatic priority | 66.47 | +0.00 | +0.00% |
| A. Semaglutide 2.4 mg | Maintenance year, Farmacenter sensitivity | S/ 16,744.00 | Broad criterion | 151.39 | -12.58 | -7.67% |
| A. Semaglutide 2.4 mg | Maintenance year, Farmacenter sensitivity | S/ 16,744.00 | Pragmatic priority | 61.37 | -5.10 | -7.67% |
| B. Naltrexone/bupropion ER | IETSI-reported annual cost | S/ 2,615.90 | Broad criterion | 23.65 | +0.00 | +0.00% |
| B. Naltrexone/bupropion ER | IETSI-reported annual cost | S/ 2,615.90 | Pragmatic priority | 9.59 | +0.00 | +0.00% |
| B. Naltrexone/bupropion ER | Arithmetic maintenance-year cost | S/ 1,971.00 | Broad criterion | 17.82 | -5.83 | -24.65% |
| B. Naltrexone/bupropion ER | Arithmetic maintenance-year cost | S/ 1,971.00 | Pragmatic priority | 7.22 | -2.36 | -24.65% |
| B. Naltrexone/bupropion ER | Arithmetic first-year titration cost | S/ 1,914.30 | Broad criterion | 17.31 | -6.34 | -26.82% |
| B. Naltrexone/bupropion ER | Arithmetic first-year titration cost | S/ 1,914.30 | Pragmatic priority | 7.02 | -2.57 | -26.82% |

*Panel A uses the primary semaglutide maintenance-year cost as reference. Panel B uses the IETSI-reported naltrexone/bupropion annual cost as reference. ER = extended release; HTA = health technology assessment; IETSI = Instituto de Evaluación de Tecnologías en Salud e Investigación.*

Figure S1. Participant flow and principal analytic denominators. Among adults aged 18 years or older, BMI was observed in 68,358 and 68,043 also had a non-missing pooled survey weight and contributed to design-weighted BMI-based estimates. Among adults aged 30 years or older, 49,011 had both a non-missing pooled survey weight and non-missing diabetes status and formed the design-weighted diabetes denominator. Diagnosed diabetes was reported by 2,599 adults, of whom 2,577 had a non-missing pooled survey weight and contributed to weighted estimates. BMI = body mass index; ENDES = Peruvian Demographic and Family Health Survey.

Figure S2. Survey-weighted candidate populations under obesity-medication and secondary cardiometabolic scenarios. Horizontal intervals are 95% confidence intervals. BMI = body mass index; CI = confidence interval; SGLT2 = sodium–glucose cotransporter 2.

Figure S3. Annual stability of survey-weighted candidate-population estimates across ENDES 2022–2024. ENDES = Peruvian Demographic and Family Health Survey; SGLT2 = sodium–glucose cotransporter 2.

Figure S4. Relationship between candidate-population size and projected annual liraglutide 3.0 mg acquisition budget at 100% coverage across progressively broader obesity-medication eligibility scenarios. BMI = body mass index.

Figure S5. Overlap among survey-observable obesity, diagnosed diabetes, and probable or treated hypertension in the adult ENDES population. Categories represent mutually exclusive combinations of these three conditions. Obesity was defined as BMI ≥30 kg/m². BMI = body mass index.

Figure S6. Annual medication-acquisition budget at 100% coverage for selected non–GLP-1 obesity-medication and secondary cardiometabolic comparator scenarios. Extended-release naltrexone/bupropion and extended-release phentermine/topiramate were costed in the pragmatic-priority obesity-medication population. Dapagliflozin and empagliflozin were costed in the diagnosed-diabetes plus risk-proxy population, and metformin was costed in adults aged 30 years or older with diagnosed diabetes. The naltrexone/bupropion bar uses the annual cost reported by IETSI-EsSalud; arithmetic maintenance-year and first-year dose-escalation sensitivities are presented in Table S10. Budgets are expressed in Peruvian soles (S/ billions). ER = extended release; GLP-1 = glucagon-like peptide-1; HTA = health technology assessment; IETSI = Instituto de Evaluación de Tecnologías en Salud e Investigación.
